# Blood inflammation relates to neuroinflammation and survival in frontotemporal lobar degeneration

**DOI:** 10.1093/brain/awae269

**Published:** 2024-08-19

**Authors:** Maura Malpetti, Peter Swann, Kamen A Tsvetanov, Leonidas Chouliaras, Alexandra Strauss, Tanatswa Chikaura, Alexander G Murley, Nicholas J Ashton, Peter Barker, Peter Simon Jones, Tim D Fryer, Young T Hong, Thomas E Cope, George Savulich, Duncan Street, William Richard Bevan-Jones, Timothy Rittman, Kaj Blennow, Henrik Zetterberg, Franklin I Aigbirhio, John T O’Brien, James B Rowe

**Affiliations:** Department of Clinical Neurosciences and Cambridge University Hospitals NHS Trust, University of Cambridge, Cambridge CB2 0SZ, UK; Department of Psychiatry, University of Cambridge, Cambridge CB2 0QQ, UK; Department of Clinical Neurosciences and Cambridge University Hospitals NHS Trust, University of Cambridge, Cambridge CB2 0SZ, UK; Department of Psychology, University of Cambridge, Cambridge CB2 3EB, UK; Department of Psychiatry, University of Cambridge, Cambridge CB2 0QQ, UK; Department of Clinical Neurosciences and Cambridge University Hospitals NHS Trust, University of Cambridge, Cambridge CB2 0SZ, UK; Department of Clinical Neurosciences and Cambridge University Hospitals NHS Trust, University of Cambridge, Cambridge CB2 0SZ, UK; Department of Clinical Neurosciences and Cambridge University Hospitals NHS Trust, University of Cambridge, Cambridge CB2 0SZ, UK; Department of Psychiatry and Neurochemistry, Institute of Neuroscience and Physiology, The Sahlgrenska Academy, University of Gothenburg, Mölndal S-431 80, Sweden; Wallenberg Centre for Molecular Medicine, University of Gothenburg, Gothenburg S-413 45, Sweden; King’s College London, Institute of Psychiatry, Psychology and Neuroscience, Maurice Wohl Institute Clinical Neuroscience Institute, London SE5 9RT, UK; NIHR Biomedical Research Centre for Mental Health and Biomedical Research Unit for Dementia at South London and Maudsley NHS Foundation, London SE5 8AF, UK; NIHR Cambridge Biomedical Research Centre, Core Biochemical Assay Laboratory, Cambridge University Hospitals NHS Foundation Trust, Cambridge CB2 0QQ, UK; Department of Clinical Neurosciences and Cambridge University Hospitals NHS Trust, University of Cambridge, Cambridge CB2 0SZ, UK; Department of Clinical Neurosciences and Cambridge University Hospitals NHS Trust, University of Cambridge, Cambridge CB2 0SZ, UK; Department of Clinical Neurosciences and Cambridge University Hospitals NHS Trust, University of Cambridge, Cambridge CB2 0SZ, UK; Department of Clinical Neurosciences and Cambridge University Hospitals NHS Trust, University of Cambridge, Cambridge CB2 0SZ, UK; Medical Research Council Cognition and Brain Sciences Unit, University of Cambridge, Cambridge CB2 7EF, UK; Department of Psychiatry, University of Cambridge, Cambridge CB2 0QQ, UK; Department of Clinical Neurosciences and Cambridge University Hospitals NHS Trust, University of Cambridge, Cambridge CB2 0SZ, UK; Department of Psychiatry, University of Cambridge, Cambridge CB2 0QQ, UK; Department of Clinical Neurosciences and Cambridge University Hospitals NHS Trust, University of Cambridge, Cambridge CB2 0SZ, UK; Department of Psychiatry and Neurochemistry, Institute of Neuroscience and Physiology, The Sahlgrenska Academy, University of Gothenburg, Mölndal S-431 80, Sweden; Clinical Neurochemistry Laboratory, Sahlgrenska University Hospital, Mölndal S-431 80, Sweden; Department of Psychiatry and Neurochemistry, Institute of Neuroscience and Physiology, The Sahlgrenska Academy, University of Gothenburg, Mölndal S-431 80, Sweden; Clinical Neurochemistry Laboratory, Sahlgrenska University Hospital, Mölndal S-431 80, Sweden; Department of Neurodegenerative Disease, UCL Institute of Neurology, London WC1N 6BG, UK; UK Dementia Research Institute at UCL, London WC1N 6BG, UK; Hong Kong Center for Neurodegenerative Diseases, Clear Water Bay, Hong Kong, China; Wisconsin Alzheimer’s Disease Research Center, University of Wisconsin School of Medicine and Public Health, University of Wisconsin-Madison, Madison, WI 53792, USA; Department of Clinical Neurosciences and Cambridge University Hospitals NHS Trust, University of Cambridge, Cambridge CB2 0SZ, UK; Department of Psychiatry, University of Cambridge, Cambridge CB2 0QQ, UK; Department of Clinical Neurosciences and Cambridge University Hospitals NHS Trust, University of Cambridge, Cambridge CB2 0SZ, UK; Medical Research Council Cognition and Brain Sciences Unit, University of Cambridge, Cambridge CB2 7EF, UK

**Keywords:** inflammation, frontotemporal lobar degeneration, blood markers, PET, survival

## Abstract

Neuroinflammation is an important pathogenic mechanism in many neurodegenerative diseases, including those caused by frontotemporal lobar degeneration. Post-mortem and *in vivo* imaging studies have shown brain inflammation early in these conditions, proportional to symptom severity and rate of progression. However, evidence for corresponding blood markers of inflammation and their relationships to central inflammation and clinical outcome are limited. There is a pressing need for such scalable, accessible and mechanistically relevant blood markers because these will reduce the time, risk and costs of experimental medicine trials. We therefore assessed inflammatory patterns of serum cytokines from 214 patients with clinical syndromes associated with frontotemporal lobar degeneration in comparison to healthy controls, including their correlation with brain regional microglial activation and disease progression.

Serum assays used the MesoScale Discovery V-Plex-Human Cytokine 36 plex panel plus five additional cytokine assays. A subgroup of patients underwent ^11^C-PK11195 mitochondrial translocator protein PET imaging, as an index of microglial activation. A principal component analysis was used to reduce the dimensionality of cytokine data, excluding cytokines that were undetectable in >50% of participants. Frequentist and Bayesian analyses were performed on the principal components to compare each patient cohort with controls and test for associations with central inflammation, neurodegeneration-related plasma markers and survival.

The first component identified by the principal component analysis (explaining 21.5% variance) was strongly loaded by pro-inflammatory cytokines, including TNF-α, TNF-R1, M-CSF, IL-17A, IL-12, IP-10 and IL-6. Individual scores of the component showed significant differences between each patient cohort and controls. The degree to which a patient expressed this peripheral inflammatory profile at baseline was correlated negatively with survival (higher inflammation, shorter survival), even when correcting for baseline clinical severity. Higher pro-inflammatory profile scores were associated with higher microglial activation in frontal and brainstem regions, as quantified with ^11^C-PK11195 mitochondrial translocator protein PET. A permutation-based canonical correlation analysis confirmed the association between the same cytokine-derived pattern and central inflammation across brain regions in a fully data-based manner.

This data-driven approach identified a pro-inflammatory profile across the frontotemporal lobar degeneration clinical spectrum, which is associated with central neuroinflammation and worse clinical outcome. Blood-based markers of inflammation could increase the scalability and access to neuroinflammatory assessment of people with dementia, to facilitate clinical trials and experimental medicine studies.

## Teaser

Malpetti *et al.* identify a pro-inflammatory profile of serum cytokines in patients with frontotemporal dementia and related conditions as compared to healthy controls. This peripheral inflammatory profile is associated with greater CNS inflammation—measured with PET—and shorter survival.

## Introduction

The pathologies of frontotemporal lobar degeneration (FTLD) cause a diverse family of clinical disorders,^1^ including the behavioural variant of frontotemporal dementia (bvFTD),^2^ the non-fluent (nfvPPA) and semantic variants of primary progressive aphasia (svPPA),^3^ right temporal variant frontotemporal dementia,^4^ progressive supranuclear palsy (PSP)^5^ and corticobasal syndrome (CBS).^6^ These FTLD-related disorders vary in their cognitive and motor features and their molecular pathology correlates but share a pressing need for new therapeutic strategies.

These disorders also have in common evidence for neuroinflammation as a pathogenic process,^7^ from preclinical,^8^ post-mortem^9-13^ and genome-wide association studies.^14-16^ The neuroinflammation manifests as microglial and astroglial activation and increased secretion of inflammatory markers, including tumour necrosis factor (TNF) and interleukin (IL) cytokines.^7^ Systemic autoimmune diseases are more prevalent in patients with FTLD-related disorders than in healthy controls or people with Alzheimer’s disease.^17,18^ Immunomodulatory and anti-inflammatory treatment strategies have been proposed to slow or prevent disease progression. However, a better understanding of the inflammatory profiles and scalable *in vivo* markers are required to inform individual prognosis, disease progression models and trial design.

One can localize and quantify neuroinflammation *in vivo* using PET. PET ligands for the mitochondrial translocator protein (TSPO), such as ^11^C-PK11195, have been implemented as *in vivo* proxies of activated microglia, showing increased cerebral inflammation in patients across all of the FTLD syndromes.^19-25^ TSPO PET has shown that higher inflammation at baseline is strongly correlated with clinical severity and predicts faster clinical decline over the following years in FTD, PSP and Alzheimer’s disease.^26-28^

It follows that immunotherapeutic strategies might reduce the risk and progression of clinical phases of FTLD-related syndromes. However, a barrier in therapeutic development and clinical trials is the lack of scalable and repeatable assays of clinically relevant neuroimmune signals. PET imaging is neither readily scalable, owing to cost and radiation dosing, nor easily accessible for many centres and countries. PET also allows one to test only a single molecular target at a time rather than capturing a comprehensive set of inflammatory markers. Fluidic markers can overcome these limitations, providing more scalable biomarkers and insight into the interaction between central and peripheral inflammatory processes.

Few clinical studies have evaluated peripheral blood markers of inflammation in patients with FTLD-related syndromes and their progression. Although blood measures do not necessarily capture the level or distribution of brain inflammation, they may nonetheless reflect risk or activity of microglia-mediated and other inflammatory cascades of relevance to experimental medicine studies. They could also enable the formulation of a more complete disease framework to assist prognosis, stratification and monitoring in trials and the identification of novel therapeutic targets. Importantly, clinically relevant and mechanistically informative blood markers for inflammation would also make research more accessible to people who lack access to major facilities or are too impaired to take part in neuroimaging-only research studies. Biochemical rather than cell-based assays would also have the advantage of simpler handling and storage for multicentre studies. However, such applications of biochemical markers of inflammation would require evidence of their level and pattern in relationship to multiple disorders and evidence of a significant relationship to cerebral inflammation.

The aim of this study was to identify clinically relevant patterns of blood-based inflammatory cytokines in people with clinical syndromes associated with FTLD. Specifically, we applied a combination of data- and hypothesis-driven approaches to determine whether serum cytokines were elevated in some or all of the FTLD-related syndromes and whether the level of serum cytokines was associated with prognosis. Based on the PET imaging literature, we predicted that markers of inflammation would be raised in each of the syndromes^19-25^ and would be associated with shorter survival.^26-28^ We then tested the secondary hypothesis that peripheral blood cytokines are correlated with inflammation of the CNS, as indexed by ^11^C-PK11195 PET.

## Materials and methods

### Participants

Two hundred and fourteen (*n* = 214) patients with clinical diagnoses associated with FTLD were recruited from specialist clinics at the Cambridge Centre for Frontotemporal Dementia and Cambridge Centre for Parkinson-plus. Exclusion criteria included recent or current acute infection, major concurrent psychiatric illness, other severe physical illness or a history of other significant neurological illness. Twenty-nine healthy controls were recruited, with Mini-Mental State Examination >26/30 and with no acute physical illness, no cognitive complaints and independent in daily function and instrumental activities of daily living.

All participants underwent phlebotomy and a standard battery of cognitive tests and questionnaires, which included the revised version of Addenbrooke’s Cognitive Examination (ACE-R)^29^ and the Revised Cambridge Behavioural Inventory (CBI-R). A sub-cohort of 44 patients (10 bvFTD, 10 nfvPPA, 7 svPPA and 17 PSP) underwent ^11^C-PK11195 PET [specifically, (*R*)-^11^C-PK11195 PET] as part of the Neuroimaging of Inflammation in Memory and Related Other Disorders (NIMROD) study.^30^

Participants with mental capacity gave their written informed consent to take part in the study. For those who lacked capacity, their participation followed the consultee process in accordance with the UK law. The research protocols were approved by the National Research Ethics Service’s East of England Cambridge Central Committee and the UK Administration of Radioactive Substances Advisory Committee.

### Blood sample collection and processing

Blood samples were obtained by venipuncture and collected in EDTA and serum gel tubes. Samples were centrifuged to isolate serum and plasma, aliquoted and stored at −70°C until further analyses. Serum cytokine analyses were carried out at the NIHR Cambridge Biomedical Research Centre, Core Biochemistry Assay Laboratory of Cambridge University Hospitals NHS Foundation Trust. The assays used the MesoScale Discovery (MSD) electrochemiluminescence immunoassay V-Plex Human Cytokine 36 plex panel and five additional cytokine assays: high-sensitivity C-reactive protein (using Siemens Dimension EXL autoanalyser), tumour necrosis factor receptor 1 (TNF-R1) and interleukin-34 (IL-34) (using MSD R-plex assays), YKL-40 (chitinase-3-like protein 1) and colony stimulating factor 1 (using MSD U-plex assays). Dilutions were made in accordance with manufacturer’s recommendations. The MSD assays were performed in duplicate, with the mean taken for the purposes of analysis. The C-reactive protein analysis was performed in singleton. Further details of the cytokine assays and markers can be found in Supplementary Table 1.

In a sub-cohort of 186 patients (44 bvFTD, 29 nfvPPA, 20 svPPA, 44 PSP and 49 CBS), plasma samples were stored at −70°C for further analyses at the Clinical Neurochemistry Laboratory in Mölndal (Sweden). Plasma samples were thawed on wet ice, then centrifuged at 500*g* for 5 min at 4°C. Calibrators (neat) and samples (plasma, 1:4 dilution) were measured in duplicate. The plasma assays measured were the Quanterix Simoa Human Neurology 4-Plex E assay [measuring amyloid-β40, amyloid-β42, glial fibrillary acidic protein (GFAP) and neurofilament light (NfL); Quanterix] and the pTau217 ALZPath assay measuring phosphorylated tau 217 (ptau217) of the human tau protein associated with Alzheimer’s disease, as previously described.^31^ Plasma samples were analysed at the same time using the same batch of reagents. A four-parameter logistic curve fit data reduction method was used to generate a calibration curve. Two control samples of known concentration of the protein of interest (high-control and low-control) were included as quality control.

### Imaging data acquisition and pre-processing

Structural MRI and ^11^C-PK11195 PET data were acquired and processed using previously described methods.^30^ Briefly, patients underwent a 3 T MRI scan, followed by a dynamic ^11^C-PK11195 PET scan for 75 min at the Wolfson Brain Imaging Centre, University of Cambridge. MRI used Siemens Magnetom Tim Trio and Verio scanners (Siemens Healthineers) with an MPRAGE T_1_-weighted sequence, and PET used GE Advance and GE Discovery 690 PET/CT (GE Healthcare) scanners. The time interval between blood sampling and PET scans was [mean ± standard deviation (SD)] 1.2 ± 2.3 months. Each T_1_ image was non-rigidly registered to the ICBM2009a template brain using ANTS (http://www.picsl.upenn.edu/ANTS/), and the inverse transform was applied to the Hammersmith Atlas (resliced from MNI152 to ICBM2009a space) to bring the regions of interest to subject MRI space. The T_1_-weighted images were segmented into grey matter, white matter and CSF with SPM12 and used to determine regional grey matter, white matter and CSF volumes and to calculate the total intracranial volume (grey matter + white matter + CSF) in each participant.

For each subject, the aligned dynamic PET image series for each scan was rigidly co-registered to the T_1_-weighted MRI image. ^11^C-PK11195 non-displaceable binding potentials (BP_ND_) were calculated in cortical and subcortical regions of interest using a modified version of the Hammersmith Atlas (www.brain-development.org), as for our previous reports.^24,26^ Prior to kinetic modelling, regional PET data were corrected for partial volume effects from CSF. Supervised cluster analysis was used to determine the reference tissue time–activity curve for ^11^C-PK11195, and BP_ND_ values were calculated in each region of interest using a simplified reference tissue model with vascular binding correction.^32^

### Statistical analyses

Statistical analyses used R v.4.1.2 (R Core Team; see specific functions in the text), JASP and MATLAB 2021b.

Age, years of education and baseline cognitive/clinical scores were compared between groups with ANOVA tests, and sex was compared with the χ^2^ test (Table 1). For frequentist tests, *P* < 0.05 after correction for multiple comparisons was considered significant [false discovery rate (FDR) correction]; uncorrected *P* < 0.05 were described for explorative analyses. For Bayesian tests, a Bayesian factor (BF) of >3 indicates positive evidence for the alternative hypothesis, and BF >10 indicates strong evidence for the alternative hypothesis, whereas BF < 0.33 and BF < 0.1 indicate positive and strong evidence, respectively, in favour of the null hypothesis.

**Table 1:** Demographic and clinical characteristics for control and patient groups

Group	*n*	Sex F/M	Age (years) Mean ± SD	ACE-R (/100) Mean ± SD	CBI-R (/150) Mean ± SD
HC	29	13/16	68.3 ± 7.9	96.2 ± 2.7	9.5 ± 8.7
bvFTD	52	22/30	63.9 ± 9.3	64.7 ± 19.2	83.1 ± 31.6
svPPA	20	8/12	68.8 ± 4.0	61.6 ± 18.7	74.5 ± 34.1
nfPPA	31	20/11	72.1 ± 7.4	69.5 ± 21.8	32.9 ± 28.0
PSP	58	29/29	69.9 ± 6.2	79.9 ± 12	49.8 ± 29.6
CBS	53	28/25	70.7 ± 7.5	73.9 ± 17.9	53.5 ± 33.9
Group difference (F)		n.s.	6.7, *P* < 0.001* (n.s. controls versus patient groups)	16.4, *P* < 0.001* (HC > all patient groups)	22.5, *P* < 0.001* (HC < all patient groups, except nfPPA)

ACE-R = revised Addenbrooke’s Cognitive Examination; bvFTD = behavioural variant frontotemporal dementia; CBI-R = Cambridge Behavioural Inventory Revised; CBS = corticobasal syndrome; F = female; HC = healthy control; M = male; nfPPA = non-fluent primary progressive aphasia; n.s. = non-significant; PSP = progressive supranuclear palsy; SD = standard deviation; svPPA = semantic variant primary progressive aphasia.

^*^Group comparisons indicate significant group effect on variance, by one-way ANOVA.

Sixteen of 41 cytokine markers were below the limit of detection for >50% participants and thus excluded from further analyses. For the remaining 25 cytokine markers, values under the threshold of detectability were replaced with the variable-specific detection threshold. Serum cytokine concentrations were log transformed, and outliers were excluded after being defined as values above or below 5 SDs from the variable-specific mean (0.2% of total 6075 cytokine values). This approach seeks to exclude extreme values attributable to methodological issues rather than pathophysiological changes. For further analyses, missing values generated by the exclusion of outliers were replaced by variable-specific means.

First, to test for dissimilarity between groups across all cytokines, values for each cytokine were averaged within group, creating a 25-cytokine vector for each group. Group-specific cytokine vectors entered a dissimilarity matrix computation analysis (R function *dist*, with the ‘Manhattan’ method), which used the absolute distance between vectors to compute the distances between the rows of the data matrix. The resulting dissimilarity matrix was included in a hierarchical cluster analysis using the complete linkage (or furthest neighbour) method.

Second, cytokine marker values were included in a principal component analysis (PCA; R function *prcomp*), with the aim of reducing dimensionality and identifing a limited number of cytokine patterns that best explain the data variance. We applied Bartlett’s test for sphericity to test the null hypothesis that there was no collinearity between the variables included in PCA and to verify that a data-reduction technique, such as PCA, can compress the data in a meaningful way. The number of components to be retained was defined by: (i) components with eigenvalues higher than one (Kaiser criterion); (ii) explained variance of >10%; and (iii) scree plot review of the ‘break’ or ‘elbow’ (Cattell criterion).

Third, to test group differences in these cytokine principal components, individual scores of the resulting components were included in a Kruskal–Wallis test with group as a factor (R function *kruskal_test*). Dunn’s *post hoc* tests were applied to compare each diagnostic group with healthy controls (R function *dunn_test*). Associations of cytokine component individual scores with clinical outcomes, and plasma markers, were tested with linear regression models, including age, sex and diagnosis as covariates. We applied both frequentist and Bayesian analysis approaches to ensure inferential robustness, allowing us to quantify evidence in favour of the null hypothesis (of no group differences).

Fourth, we investigated the relationship between cytokine-derived component scores and survival. Survival data were available for 207 of the patients (140 deceased and 67 still alive; 48 bvFTD, 53 CBS, 31 nfPPA, 55 PSP and 20 svPPA), at the census date 17 October 2023. Survival analysis used Cox proportional hazards regression (R function *coxph*), including cytokine component scores and years from blood test to death as variables of interest and with age, sex and diagnosis group as covariates. A further explorative analysis was performed in a subgroup of 133 participants (77 deceased) to accommodate baseline severity, including baseline plasma NfL levels, duration of symptoms (time interval between symptom onset and blood sampling) and ACE-R scores.

Fifth, we tested the association between cytokine-derived component individual scores and plasma levels of NfL, GFAP and pTau217. We applied Spearman’s correlation and multiple regression models with each of the three plasma markers of interest as dependent variables and with cytokine-derived component individual scores, in addition to age, sex and diagnosis as independent variables. Bayesian Spearman’s correlation (https://osf.io/gny35/) was applied secondarily to quantify evidence in favour of the null hypothesis (of no associations).

Finally, we tested the association between cytokine-derived component individual scores and central inflammation, quantified as regional ^11^C-PK11195 PET binding potential. Based on previous publications in patients with FTD^20,26^ and PSP,^24,28^ regional ^11^C-PK11195 PET values were averaged within five hypothesis-driven regions of interest representing areas of peak neurodegenerative pathology: bilateral frontal and temporal lobes, and brainstem. A multiple regression model was fitted to examine the individual ability and the combined ability to explain variance in cytokine-derived component individual scores using regional values of central inflammation, in addition to age, sex and diagnosis as independent variables. The model used stepwise backward selection (entry criterion α = 0.05 and elimination criterion α = 0.1) to identify the optimal model.

To confirm our results and the association between peripheral signatures of inflammation and microglial activation in the CNS, beyond our hypothesized regions of interest, we examined the relationship between serum cytokines and whole-brain regional ^11^C-PK11195 PET values with a two-level analytical approach.^33-35^ In the first-level analysis, we assessed the multidimensional brain–cytokine relationships using permutation-based canonical correlation analysis (CCA). This analysis described the linear relationship between the two multivariate datasets^36^ that were mapped to latent, common factors, or canonical variates, underlying these associations.^34^ Namely, Dataset 1 consisted of the 25 cytokines in the previous analysis. Dataset 2 included the 89 Hammersmith-based regional ^11^C-PK11195 PET binding potentials. For this approach, instead of replacing missing cytokine values by variable-specific means, we imputed values under fully conditional specification, using the default settings of the multivariate imputation by chained equations (MICE) in R. Should the cytokine-derived component be similar to the one obtained with the first approach, this would ensure that results were not driven by a specific replacement approach. All variables were then standardized into *z*-scores before CCA. The CCA was permuted 5000 times to determine significance and ensure stability of the final components. Next, we tested whether the identified cytokine-relevant canonical variate of brain inflammation remained significant after correcting for age, sex and diagnosis, using a second-level linear regression analysis.

## Results

Two hundred and fourteen patients with clinical diagnoses associated with FTLD and 29 cognitively unimpaired controls were recruited. Fifty-two patients met diagnostic criteria for bvFTD,^2^ 51 for primary progressive aphasia, comprising 31 cases of nfvPPA and 20 of svPPA,^3^ 58 for PSP^5^ and 53 for CBS.^6^ Patients were age- and sex-matched with controls (Table 1). As expected, patients with bvFTD were, on average, younger than patients with nfPPA, PSP and CBS. Controls had higher ACE-R scores and lower CBI-R scores than patient groups. Patients with PSP had higher ACE-R scores than patients with bvFTD and svPPA. Controls had lower CBI-R scores than all patient groups, except for nfPPA.

### Group differences in the pattern of cytokine values

**Figure 1:**
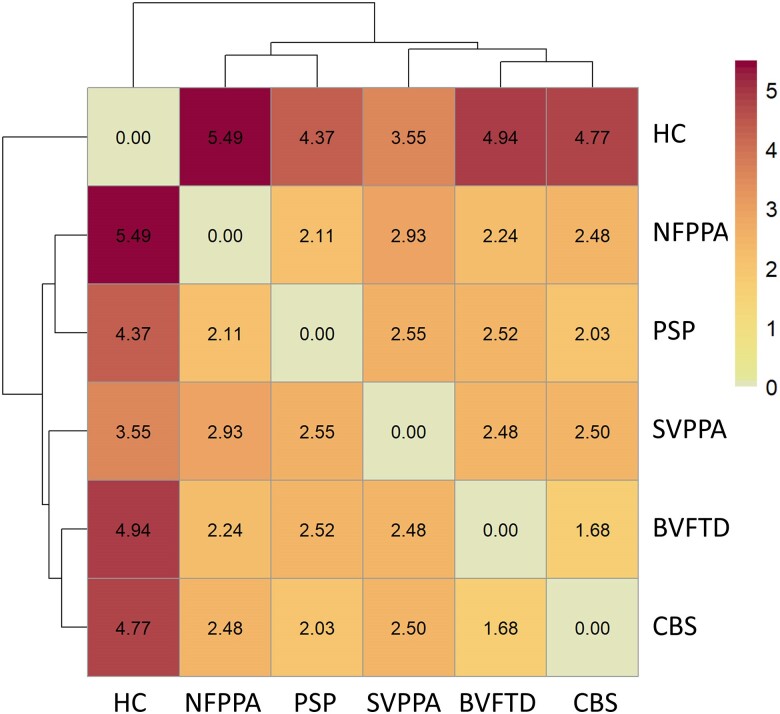
**Dissimilarity between groups across all cytokines.** Darker colours and larger values indicate greater distance between groups, whereas lighter colours indicate relative similarity. bvFTD = behavioural variant of frontotemporal dementia; CBS = corticobasal syndrome; HC = healthy controls; NFPPA = non-fluent primary progressive aphasia; PSP = progressive supranuclear palsy; SVPPA = semantic variant of primary progressive aphasia.

Group-based dissimilarity analyses across 25 cytokines and the related hierarchical cluster complete-linkage analysis identified greater distance values between controls and each patient group than between diagnostic groups (Fig. 1). The smallest distance value was found between PSP and CBS groups and between bvFTD and CBS groups. The largest distance between patient groups was found between svPPA and nfPPA. In other words, the relative distribution of cytokine concentrations, not the magnitude of their concentration, differentiated all patient groups with an FTLD disorder from controls.

### Cytokine-derived principal components: cytokine profiles

**Figure 2:**
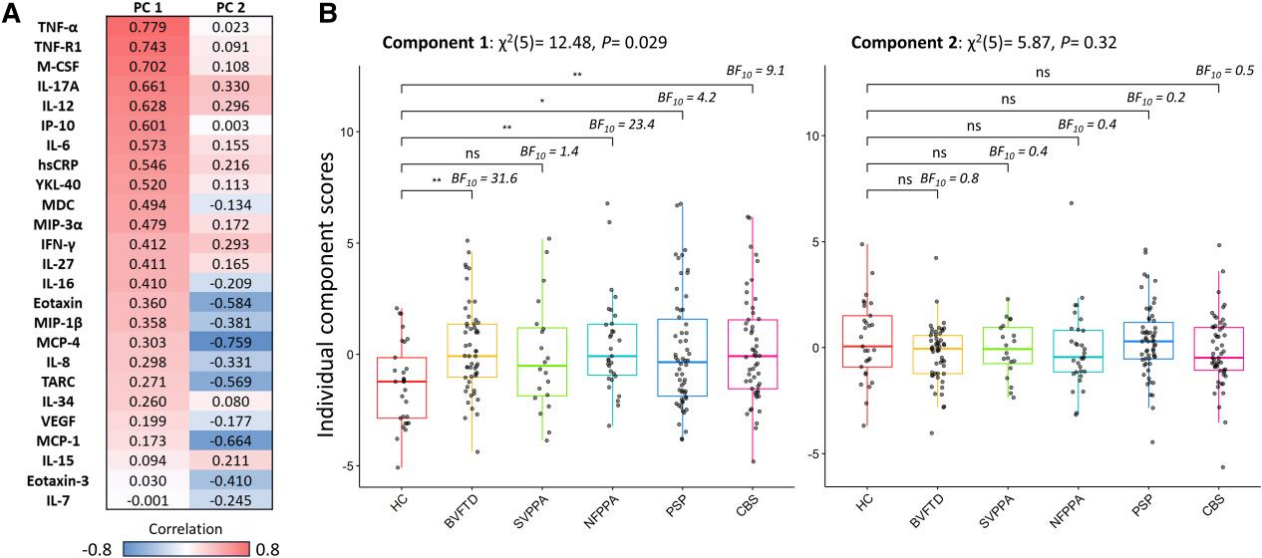
**Cytokine-derived principal components (PC) and group comparisons.** (**A**) Contribution of each cytokine to Component 1 and Component 2; colours represent the strength and direction of the associations. (**B**) Group comparisons assessed by Kruskal–Wallis one-way ANOVA and Dunn’s *post hoc* tests (*P*-values: ***0.001, **0.01, *0.05). BF10 = Bayes factor in favour of the alternative hypothesis and against the null; bvFTD = behavioural variant of frontotemporal dementia; CBS = corticobasal syndrome; HC = healthy controls; NFPPA = non-fluent primary progressive aphasia; PSP = progressive supranuclear palsy; SVPPA = semantic variant of primary progressive aphasia.

The highly significant Bartlett’s test indicated that the cytokine data were suitable for PCA (χ^2^ = 856.6, *P* < 0.00001). The Kaiser–Meyer–Olkin (KMO = 0.79) criterion also indicated the suitability for PCA. Applying the three criteria described in the ‘Materials and methods’ section, the first two components were selected for further analyses. Component 1 and Component 2 explained 21.5% and 11.0% of the variance, respectively. Component 1 was positively loaded mainly onto TNF-α, TNF-R1, macrophage colony-stimulating factor or colony stimulating factor 1 ( M-CSF), IL-17A, IL-12, interferon γ-induced protein 10 kDa (IP-10) and IL-6, whereas Component 2 was negatively weighted mainly by monocyte chemoattractant protein-4 (MCP-4), monocyte chemoattractant protein-1 (MCP-1), eotaxin and thymus- and activation-regulated chemokine (TARC) (see Fig. 2A for contribution of each cytokine). Protein–protein interactions and pathways of the lead cytokines (with contribution values of >0.4) of Component 1 were explored with the STRING software (https://string-db.org/),^37^ which systematically integrates protein–protein physical interactions and functional associations from published literature (Supplementary Fig. 1). Similar components and individual component loadings were obtained when excluding participants with high-sensitivity C-reactive protein > 10 mg/l (*n* = 17), which might be indicative of either central or peripheral inflammatory conditions (Supplementary Fig. 2).

### Patient groups show elevated inflammation

Kruskal–Wallis one-way ANOVA on Component 1 identified significant group differences (12.48, *P* = 0.029). Dunn’s multiple comparison *post hoc* test highlighted significant differences between controls and each diagnostic group (controls versus bvFTD: 3.12, *P* = 0.002; nfPPA: 3.03, *P* = 0.003; PSP: 2.32, *P* = 0.021; CBS: 2.78, *P* = 0.005), except for patients with svPPA (1.72, *P* = 0.086) (see Fig. 2B and Supplementary Table 2 for pairwise comparisons). Kruskal–Wallis one-way ANOVA on Component 2 did not identify any significant group differences (Fig. 2B). Bayesian pairwise *post hoc* tests confirmed the results obtained with the frequentist approach (see Fig. 2B for BF).

### Inflammation is associated with biomarkers of neurodegeneration

The peripheral blood cytokine profile was correlated with biomarkers of neurodegeneration. Specifically, the univariate correlations between cytokine-derived Component 1 individual scores and plasma markers identified weak but positive associations with plasma levels of NfL (ρ = 0.113, *P* = 0.06, BF = 0.7), GFAP (ρ = 0.159, *P* = 0.015, BF = 3.2) and pTau217 (ρ = 0.145, *P* = 0.025, BF = 3.9) across all patients (Supplementary Fig. 3). Multiple regression models with age, sex and diagnosis as covariates confirmed the association between cytokine profile Component 1 and NfL (*F* = 4.43, *P* = 0.037), GFAP (*F* = 4.76, *P* = 0.030) and pTau217 (*F* = 4.15, *P* = 0.043). Bayesian correlation results highlighted the weakness of the evidence for these associations (1 < BF < 3). Univariate correlations and regression models did not identify statistically significant associations between cytokine-derived Component 2 individual scores and plasma markers (*P* > 0.05).

### Higher inflammation indicates worse prognosis

**Figure 3:**
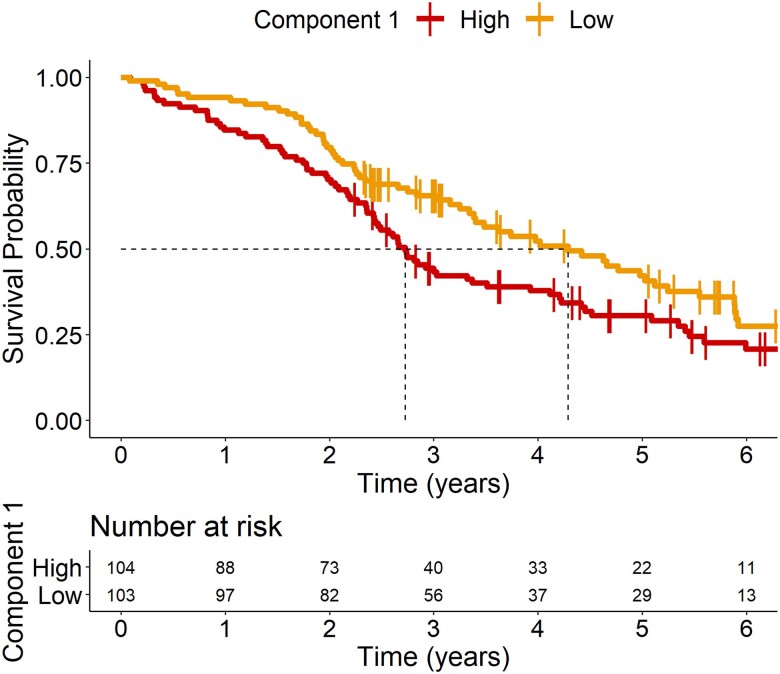
**Kaplan–Meir survival curve of loadings on Component 1.** Patients were separated into two groups based on their loading onto Component 1, with high loadings being greater than or equal to the median (≥−0.180) and low loadings being lower than the median (<−0.180).

We tested Component 1 as a prognostic biomarker (in patients only) using Cox proportional hazards regression with cytokine Component 1 individual scores and days from blood test to death as predictors of interest for survival and with age, sex and disease groups as covariates. The individual participant loadings on Component 1 were significantly associated with time to death [hazard ratio 1.1 (1.02–1.19), *P* = 0.011] (see Table 2, ‘basic model’). To illustrate this effect, we plotted separately the patients with high and low values on this component, as separated by the median (Fig. 3). When adding baseline plasma NfL, symptom duration and ACE-R scores to the model as proxies of disease severity, the predictive value of Component 1 on survival remained significant [hazard ratio 1.14 (1.02–1.27), *P* = 0.0199] (Table 2, ‘model with severity’).

**Table 2:** Cox proportional hazards model of time from blood sample to death

		HR	CI Lower 95%	CI Upper 95%	SE	*z*	Pr(>|*z*|)
Basic model (*n* = 207)							
Component 1	**PC1**	**1.101**	**1.022**	**1.185**	**0.038**	**2.533**	**0.011**
Model with covariates (*n* = 207)							
Component 1	**PC1**	**1.083**	**1.003**	**1.170**	**0.039**	**2.032**	**0.042**
Age		1.060	1.032	1.088	0.014	4.236	<0.001
Sex	Female (reference)						
	Male	1.127	0.797	1.593	0.177	0.677	0.499
Diagnosis	BVFTD (reference)						
	CBS	1.206	0.711	2.047	0.270	0.696	0.487
	NFPPA	0.516	0.270	0.987	0.330	−2.000	0.045
	PSP	1.409	0.846	2.348	0.260	1.317	0.188
	SVPPA	0.466	0.232	0.936	0.355	−2.145	0.032
Model with severity (*n* = 133)							
Component 1	**PC1**	**1.138**	**1.021**	**1.269**	**0.056**	**2.328**	**0.0199**
Age		1.078	1.032	1.127	0.023	3.356	0.001
Sex	Female (reference)						
	Male	0.835	0.504	1.386	0.258	−0.697	0.486
Diagnosis	BVFTD (reference)						
	CBS	1.603	0.645	3.988	0.465	1.016	0.310
	NFPPA	0.759	0.295	1.956	0.483	−0.571	0.568
	PSP	3.541	1.439	8.716	0.459	2.752	0.006
	SVPPA	0.439	0.145	1.325	0.564	−1.460	0.144
Severity	Plasma NfL	1.002	0.999	1.004	0.001	1.521	0.128
	Symptom duration	0.911	0.816	1.017	0.056	−1.661	0.097
	ACE-R	0.986	0.972	1.001	0.007	−1.884	0.060

The contribution of the cytokine-derived Component 1 scores is highlighted in bold in each model. bvFTD = behavioural variant frontotemporal dementia; CBS = corticobasal syndrome; CI = confidence interval; HR = hazard ratio; NfL = neurofilament light; nfPPA = non-fluent primary progressive aphasia; PSP = progressive supranuclear palsy; SE = standard error; svPPA = semantic variant primary progressive aphasia.

### Peripheral blood and brain PET markers of inflammation are correlated

**Figure 4:**
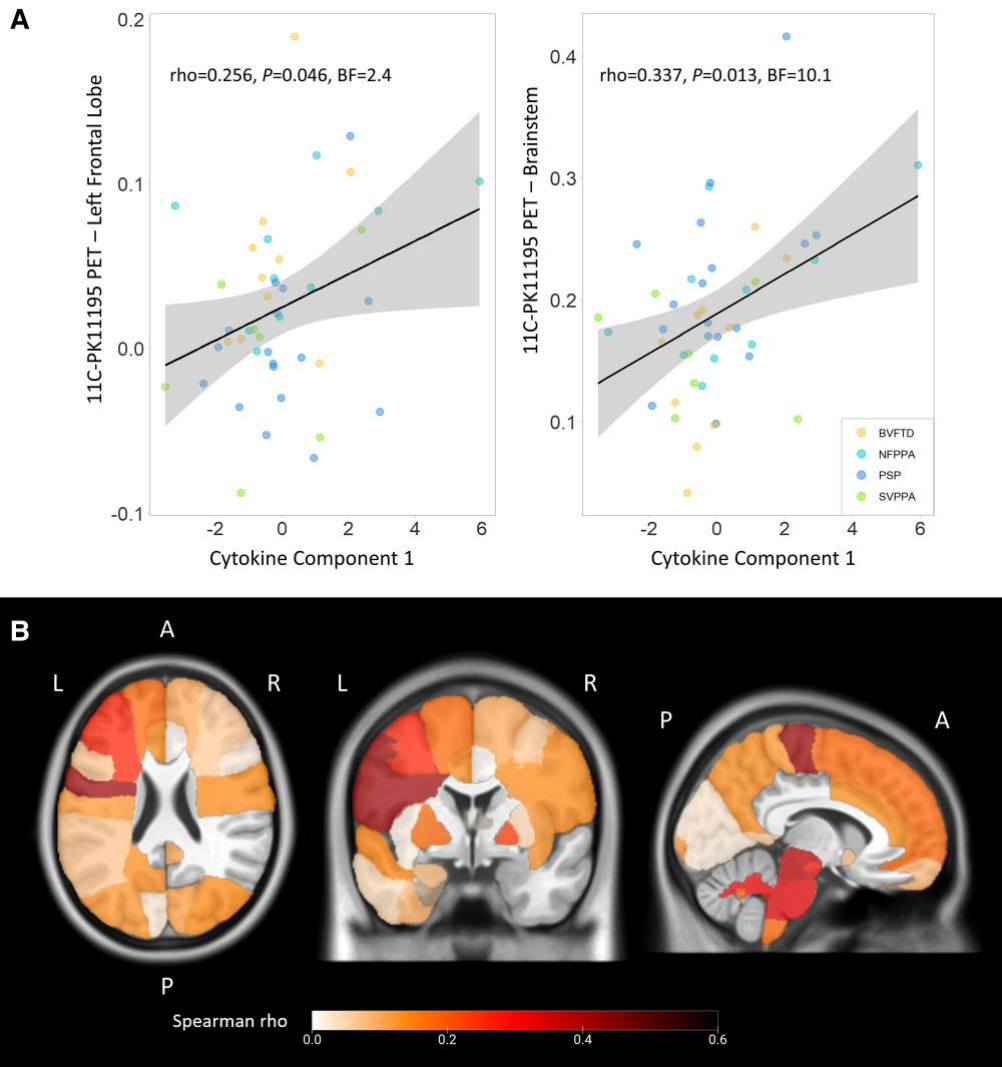
**Associations between peripheral and central inflammation.** (**A**) Correlations between cytokine-derived Component 1 individual scores and ^11^C-PK11195 BPND in left frontal lobe and brainstem, which were identified as significantly associated with Component 1. (**B**) Positive correlation coefficients (Spearman’s ρ) from explorative regional analyses between cytokine-derived Component 1 individual scores and regional ^11^C-PK11195 binding potentials across the whole brain. A = anterior; L = left; P = posterior; R = right.

Multivariate regression models on cytokine-derived component individual scores used regional values of ^11^C-PK11195 PET (marker of central inflammation), in addition to age, sex and diagnosis as independent variables. The ANOVA of the models identified levels of inflammation in the left frontal lobe (estimate = 7.89, *t*-value = 1.12, *F* = 4.75, *P* = 0.036) and in the brainstem (estimate = 9.698, *t*-value = 2.34, *F* = 6.50, *P* = 0.015) as statistically significant and positively associated predictors of cytokine-derived Component 1 individual scores. The other regional values, age, sex and diagnosis were not statistically significant. After stepwise backward selection, the final model of multiple regression on cytokine-derived Component 1 (adjusted *R*^2^ = 0.188; *P* = 0.005) included inflammation regional values in the left frontal lobe (estimate = 7.85, *t*-value = 1.81, *F* = 5.19, *P* = 0.028) and brainstem (estimate = 9.02, *t*-value = 2.61, *F* = 6.79, *P* = 0.0127) as predictors. Univariate associations between each significant predictor and individual scores of cytokine-derived Component 1 are described in Fig. 4A, with frequentist and Bayesian Spearman’s correlation results. Exploratory correlation analyses were performed between regional ^11^C-PK11195 binding potentials across the whole brain and cytokine-derived Component 1 individual scores (Fig. 4B; see Supplementary Fig. 4 for the numbers and names of corresponding regions in the Hammersmith Atlas). Significant positive correlations were found with the left middle frontal gyrus (ρ = 0.262), the left orbitofrontal cortex (ρ = 0.332), the left precentral gyrus (ρ = 0.420), the midbrain (ρ = 0.375) and pons (ρ = 0.324), and white matter of the cerebellum (ρ = 0.314; *P* < 0.05).

**Figure 5:**
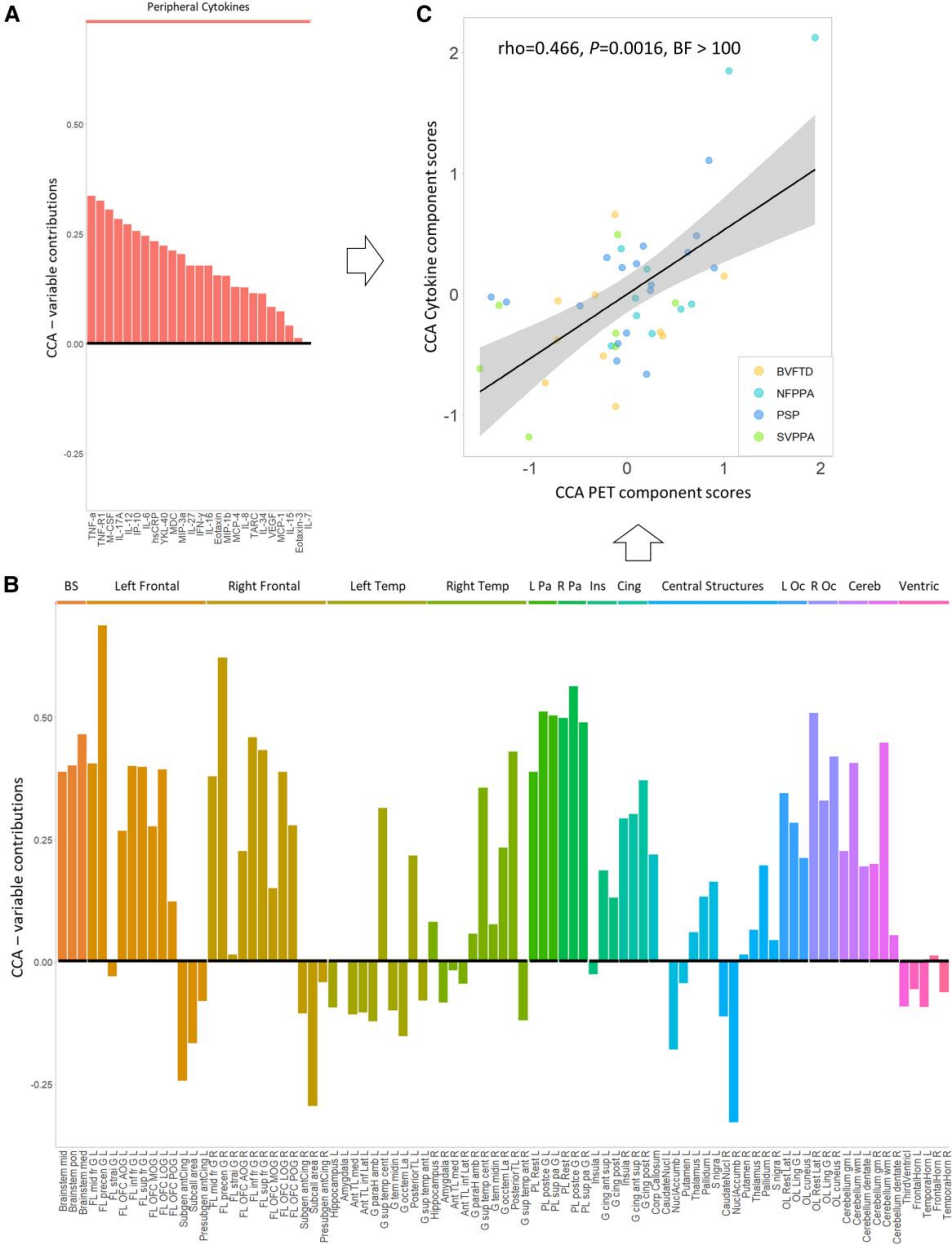
**Comfirmatory canonical correlation analysis (CCA) on cytokines and TSPO PET regional values.** The bar graph shows contribution loadings for each variable to the first CCA component, which combines serum cytokines (**A**) and ^11^C-PK11195 PET BPND regional values (**B**). Panel **C** shows the positive correlation (Spearman’s ρ) between cytokine-derived (*y*) and PET-derived (*x*) CCA individual scores. BS = brainstem; Cereb = cerebellum; Cing = cingulate; Ins = insula; L/R Oc = left/right occipital; L/R Pa = left/right parietal; Temp = temporal; Ventric = ventricles. See Supplementary Table 3 for a full list of loadings.

Finally, we used CCA to test the association between cytokine-derived patterns and central brain inflammation in a fully data-driven approach. This revealed one significant component (all groups *r* = 0.417, *P* = 0.036). This component indicated a positive association between a pattern of cytokines loaded onto TNF-α, TNF-R1, M-CSF, IL-17A, IL-12, IP-10 and IL-6 (Fig. 5A, similar to Component 1 in Fig. 2A) and a pattern of microglial activation mainly weighted by PET signal in the brainstem, frontal and parietal regions, but also by cerebellar white matter and lateral occipital regions (Fig. 5B; see Supplementary Table 3 for a full list of loadings from cytokine and regional TSPO PET values on the first CCA component). Higher individual scores in the cytokine-derived component were associated with higher individual scores in the PET-derived component (ρ = 0.466, *P* = 0.0016, BF = 2.86 × 10^44^), even after correcting for age, sex and diagnosis (estimate = 0.41, *t*-value = 3.19, *F* = 23.7, *P* < 0.0001; Fig. 5C).

## Discussion

This study identified a clinically relevant profile of pro-inflammatory serum cytokines across FTLD-related conditions, which differentiates patient groups from controls and is expressed across all clinical syndromes. The inflammatory cytokine pattern was positively associated with higher levels of cerebral microglial activation on ^11^C-PK11195 TSPO PET imaging. The degree to which a patient expressed this peripheral inflammatory profile was correlated with survival. We suggest that blood-based markers of inflammation could increase the scalability and access to neuroinflammatory assessment of people with dementia, to facilitate clinical trials and experimental medicine studies.

Recent advances in blood markers of *in vivo* inflammation in other forms of dementia^38-40^ have raised the ambition for FTLD-related conditions. Although fluid markers confirmed abnormally high levels of inflammation in patients with FTLD-related conditions^41-46^ (for a review, see Bright *et al*.^7^), they have been largely confined to CSF, small case reports, limited panels and/or single markers. CSF markers can be highly sensitive, but unlike PET imaging they do not reveal the regional distribution of inflammation, despite being invasive and of limited repeatability and patient acceptability. Instead, there is the need for clinically relevant and mechanistically informative blood markers of neuroinflammation to accelerate drug discovery and trial participation. Here, we investigated the covariance of serum cytokines to define inflammatory profiles with data-driven approaches. We validated the clinical relevance of these profiles by testing for associations with markers of neurodegeneration (including NfL), brain inflammation (PET) and clinical progression (survival).

The most prominent inflammatory profile, identified by our approach with both PCA and CCA, was driven by pro-inflammatory cytokines, including TNF-α, TNF-R1, M-CSF, IL-17A, IL-12, IP-10 and IL-6, with common pathways and physical/functional interactions as part of an immune response (Supplementary Fig. 1). This multivariate (multi-cytokine) pattern supports and expands the results of more selective prior studies. TNF-α and its receptors regulate a large range of physiological functions, including immune reactions to infections, cell death induction and immune surveillance, and play a central role in initiating and regulating the cytokine cascade during an inflammatory response. Several studies have described elevated TNF-α levels in patients with Alzheimer’s disease (see metanalyses^47,48^). In patients with Alzheimer’s disease, increased serum TNF-α levels were found to be associated with an increased rate of cognitive decline.^49^ When TNF-α signalling is inhibited both in patients with Alzheimer’s disease and in transgenic mouse models, Alzheimer’s pathology is attenuated and cognition improves. Epidemiological studies suggest that blocking TNF-α might reduce risk for Alzheimer’s disease in those with autoimmune conditions^50,51^; whereas the upregulation of TNF-α leads to the exacerbation of pathology.^52^ However, studies on TNF-α in FTLD cohorts are limited. A recent study evaluated plasma levels of six cytokines in 39 patients with bvFTD; higher levels of TNF-α, among other markers, were associated with clinical severity and brain atrophy in frontal–limbic–striatal regions.^53^ The relevance of TNF-α in FTLD-related conditions was further confirmed by evidence from a large cohort of patients with pathogenic C9orf72, progranulin or *MAPT* variants, showing that higher levels of TNF-α at baseline are associated with faster clinical progression and more rapid brain atrophy rates longitudinally.^54^ In addition, plasma TNF-α improved the prediction of asymptomatic-to-symptomatic conversion, beyond plasma NfL alone.^54^ Elevated TNF-α levels were also previously observed in plasma of 129 patients with svPPA compared with neurologically healthy controls.^17^ Our study expands this series of blood-based marker studies, investigating a larger panel of inflammatory markers in patients with FTD, PPA, PSP and CBS, recruited in clinically and epidemiologically based research studies. Collectively, the data suggest that pro-inflammatory peripheral profiles, especially driven by TNFα, might contribute to FTLD disease progression and inform disease prognosis, in both sporadic and genetic forms of the disease.

Beyond TNF-α, previous studies on blood-based inflammation biomarkers in FTLD reported an increase of plasma IL-6 levels in 230 patients with FTD, irrespective of the clinical and genetic disease subtype,^55^ in addition to increased serum levels of IL-6 in 14 carriers of *GRN* mutations.^56^ Studies on serum/plasma levels of M-CSF, IL-17A, IL-12 and IP-10 in patients with FTLD are limited or inconclusive. In contrast to previous studies, our approach combines a large panel of serum cytokines with data-driven methods to define inflammatory profiles and includes patients from a large range of clinical syndromes and diseases related to FTLD. This approach might provide a comprehensive and complementary framework of inflammatory pathways and immune interactions rather than the application of single markers and hypothesis-driven analysis in single-disease cohorts. Importantly, previous PET imaging studies showed that inflammation is a common feature across all FTLD-associated clinical conditions, with the regional distribution of neuroinflammation being syndrome specific.^20,23-25^ With the advantages of moving to blood-based markers for scalability, inclusivity and repeatability, we lose the information on brain localization that comes with imaging. Distinct from PET, with its preservation of topography, our data-driven approach to blood markers (PCA and CCA methods) identified a cytokine-based inflammatory profile that is not syndrome specific. This means that cytokines panels are unlikely to help differential diagnosis between FTLD-associated syndromes. Despite this limitation, a potential advantage of the transdiagnostic cytokine profile is that an effective immunotherapeutic approach to one syndrome might be applied more readily across all syndromes.

The cytokine-derived pro-inflammatory profile (Component 1), in addition to differentiating patient groups from healthy controls, showed prognostic value. Individuals with higher scores in expressing this inflammatory profile were associated with subsequent faster decline and shorter time from blood sampling to death, over and above age, sex and diagnosis and indexes of disease baseline severity (NfL levels, symptom duration and cognitive performance). In particular, here we considered survival rate to test for the prognostic utility of inflammatory profiles, because the high clinical heterogeneity across the FTLD spectrum and measurement ceiling effects make it difficult to capture clinical progression with a single cognitive test or questionnaire over time. In line with our results, genome-wide association studies implicated inflammatory pathways in the aetiology and progression of FTLD-related conditions. For example, associations were found between a common variation at the leucine-rich repeat kinase 2 (*LRRK2*) locus and survival from symptom onset to death in patients with PSP, which might be mediated by the effect of increased LRRK2 expression in microglia pro-inflammatory responses.^15^ Plasma NfL has been highlighted as a promising marker for disease progression and as a useful trial end point in patients with FTLD.^57-59^ Adding NfL levels to the survival model resulted in the cytokine-derived component retaining its predictive value. This suggests that combining biomarkers of neuronal damage and inflammation, such as TNF-α levels, might improve patient-specific prognostic estimations. This aligns with our previous studies showing that neuroinflammation levels, as measured with TSPO PET, provide additional predictive information on clinical and cognitive decline across the FTLD, over and above measures of brain atrophy, such as structural MRI.^26,28^

Importantly, we found that pro-inflammatory cytokine profiles (i.e. Component 1) identified in serum samples of patients with FTLD-related conditions are positively associated with higher levels of microglial activation in FTLD-specific brain regions. The association between the cytokine-derived component and central inflammation was confirmed with multiple approaches, including hypothesis-based frequentist and Bayesian correlation analyses and the fully data-driven CCA. Specifically, the latter confirmed a strong association between pro-inflammatory cytokines driving Component 1 and a composite of brain inflammation across the whole brain. Although blood-based quantification of inflammatory protein levels does not capture entirely the central inflammation processes happening in the brain, peripheral and central immune responses are most likely to engage in bidirectional interactive processes. Putative mechanistic linkages between peripheral and central immune interaction have been proposed: direct pathways by which peripheral immune cells might directly infiltrate the CNS; in addition to indirect pathways by which systemic inflammation could drive a chronic modulation of microglial function.^60^ Post-mortem studies reveal increased numbers of activated microglia and other neuroinflammatory responses in disease-specific regions that herald and topographically mirror neurodegeneration,^9,12,13,61^ including T-cell infiltration in the brain and increased cytokine concentrations. TNF-α is synthesized by microglial cells following activation, in addition to peripheral blood mononuclear cells, and can induce the activation of additional resting microglial cells or astrocytes. Along with TNF-α, interferon gamma, which is a potent stimulator of other inflammatory cytokines, and YKL-40 also significantly contributed to the cytokine-derived Component 1. Interferon gamma from both CD4^+^ and CD8^+^ T cells promotes astrocyte proliferation and activation, resulting in exacerbated neuroinflammation. Increased interferon gamma expression is associated with T-cell brain infiltration and activated microglia with increased blood–brain barrier permeability.^62^ YKL-40 is also upregulated in several neurological disorders, and during neuroinflammatory processes it is overexpressed in reactive astrocytes and microglial cells.^63^ The association between pro-inflammatory cytokine levels and regional microglial activation suggest that blood-based biomarkers of inflammation might be able to capture inflammatory processes that are relevant for central inflammation and brain changes. Studies linking peripheral and central inflammation in neurological diseases are relatively sparse. For example, increased TSPO PET binding has been linked to elevated peripheral inflammatory markers in patients with multiple risk factors for stroke (e.g. C-reactive protein)^64^ and patients with Parkinson’s disease (e.g. IL-1β, interferon gamma and IL-6).^65^ However, in patients with mild cognitive impairment attributable to Alzheimer’s disease, blockade of peripheral TNF-α with peripheral inhibitors, such as etanercept, does not show statistically significant effects on microglial activation as measured by ^11^C-PK11195 PET, although the sample size in that study was limited (treatment *n* = 6, placebo *n* = 4).^66^ Further mechanistic studies are needed to clarify the crosstalk between peripheral and central immune systems in FTLD and related diseases and to determine whether this interaction might represent a valid therapeutic target. Longitudinal clinical studies, including early phases of the disease, are also needed to clarify changes in the central–peripheral immune dynamics along the disease course.

Our study has limitations. Patients were included according to clinical diagnosis: post-mortem confirmation of pathology is available so far in only a small number of cases. This requires caution in the interpretation of likely underlying molecular pathology, especially in clinical syndromes with poor clinical–pathological correlation, such as cases of CBS^67^ or two similarly prevalent alternative pathologies like bvFTD (Tau versus TDP-43). Co-pathology might also occur, especially in older adults with age-related increase in the prevalence of Alzheimer pathology. Statistical power also needs consideration. Patients with svPPA overall showed higher absolute values of cytokine-derived Component 1 in comparison to controls (Fig. 2), in line with previous reports describing elevated plasma TNF-α levels in large cohorts of patients with svPPA (*n* = 129).^17^ The magnitude of the group difference is similar for svPPA to that for for other syndromes. However, in our study, group differences between the svPPA and control cohorts were not significant (*P* = 0.086). This is likely to be a type II error. We included a large sample size of the patient with FTLD, leading to well-powered analyses, but the sub-cohort of patients with svPPA had a smaller sample size (*n* = 20). The BF was in favour of a group difference and not in favour of the null, but at BF = 1.4 the evidence is anecdotal or weak, rather than strongly positive. Future studies on svPPA with larger cohorts might be needed to identify statistically significant differences in cytokine levels. CSF samples were available in a small group of patients (*n* < 10), thus CSF–blood correlations were not achievable with meaningful power. However, we included TSPO PET as measure of brain inflammation to validate the relevance of the cytokine-derived pro-inflammatory pattern for central inflammatory processes. Although TSPO PET is the most widely used biomarker to quantify *in vivo* central inflammation in patients with neurodegenerative diseases, its signal reflects TSPO overexpression in activated microglia cells and cannot fully capture the complexity of neuroinflammation in these conditions. Ongoing studies are testing the PET-to-autopsy associations to validate the utility of TSPO PET and determine the cellular specificity of the ligand binding. Blood-based inflammatory markers are not disease specific and might capture co-morbidities that are not directly linked to dementia. Co-occurring inflammatory conditions or the use of specific anti-inflammatory medication are unlikely to account for our results for several reasons: (i) at recruitment, the NIMROD source study excluded patients with co-morbid pro-inflammatory conditions, such as rheumatoid arthritis, inflammatory bowel disease, psoriasis or other autoimmune disorders, and cancer; (ii) all contributory cohorts excluded recent systemic infections and current medical illness; (iii) we found strong correlations between the blood-based inflammation profile and regional TSPO PET; and (iv) some of the lead cytokines loaded onto Component 1 have been shown to be produced by activated microglia and/or astrocytes or can directly influence their immune response. Moreover, the presence of a limited number of patients with elevated high-sensitivity C-reactive protein was not driving the cytokine-derived profile (Supplementary Fig. 2). Taken together, these factors provide reassurance that the blood-based inflammation markers relate in large part to brain inflammatory changes in FTLD-associated syndromes. Our cohort was predominantly white/Caucasian, reflecting the ethnicity distribution of the >65-year-old population in the UK (94% ‘white’ in the 2021 national census), and further studies are needed to test the generalization of results to other racial groups and to identify environmental, genetic and socioeconomical factors that might influence the immune responses in dementia. Finally, the inflammatory markers were measured at a single time point, whereas longitudinal studies would be needed to test for disease-related dynamics of inflammation.

## Conclusion

In conclusion, our data-driven approach identified a pro-inflammatory profile across each of the major FTLD-related conditions, which is positively associated with worse survival outcome and higher levels of brain microglial activation. Our results indicate the relevance of peripheral markers of inflammation to predict clinical progression in patients across the FTLD spectrum. Blood-based tests could greatly increase the scalability and access to neuroinflammatory assessment in dementia and experimental medicine studies. The combination of blood-based biomarkers for inflammation, along with markers for neurodegeneration (i.e. NfL) to evaluate patients might be a valuable approach to stratify patients into those likely to exhibit slow versus fast decline, improving cohort selection for clinical trials and the interpretation of clinical outcomes.

## Supplementary Material

awae269_Supplementary_Data

## Data Availability

Anonymized processed data can be shared upon request to the corresponding or senior authors. Raw data may also be requested but are likely to be subject to a data transfer agreement with restrictions required to comply with participant consent and data protection regulations.
