# Supplementary material for: Blood inflammation relates to neuroinflammation and survival in frontotemporal lobar degeneration
Source: Brain. 2024 Aug 19;148(2):493–505. doi: 10.1093/brain/awae269 (PMC7617268; doi:10.1093/brain/awae269)
Supplement: awae269_Supplementary_Data [file awae269_supplementary_data.pdf]

Blood inflammation relates to neuroinflammation and survival in frontotemporal lobar degeneration

Maura Malpetti[1], Peter Swann[2], Kamen A Tsvetanov[1,2], Leonidas Chouliaras[3], Alexandra Strauss[1], Tanatswa Chikaura[1], Alexander G Murley[1], Nicholas J. Ashton[4,5,6,7], Peter Barker[8], P Simon Jones[1], Tim D. Fryer[1], Young T. Hong[1], Thomas E Cope[1,9], George Savulich[2], Duncan Street[1], W Richard Bevan-Jones[2], Timothy Rittman[1], Kaj Blennow[4,10], Henrik Zetterberg[4,10,11,12,13,14], Franklin I. Aigbirhio[1], John T. O'Brien[2,*] and James B. Rowe[1,9,*]

1 University of Cambridge Department of Clinical Neurosciences and Cambridge University Hospitals NHS Trust, Cambridge, United Kingdom
2 Department of Psychology, University of Cambridge, Cambridge, United Kingdom
3 Department of Psychiatry, University of Cambridge, Cambridge, United Kingdom
4 Department of Psychiatry and Neurochemistry, Institute of Neuroscience and Physiology, The Sahlgrenska Academy, University of Gothenburg, Mölndal, S-431 80, Sweden
5 Wallenberg Centre for Molecular Medicine, University of Gothenburg, Gothenburg, S-413 45, Sweden
6 King's College London, Institute of Psychiatry, Psychology and Neuroscience, Maurice Wohl Institute Clinical Neuroscience Institute, London, SE5 9RT, UK
7 NIHR Biomedical Research Centre for Mental Health and Biomedical Research Unit for Dementia at South London and Maudsley NHS Foundation, London, SE5 8AF, UK
8 NIHR Cambridge Biomedical Research Centre, Core BiochemicalAssay Laboratory, Cambridge University Hospitals NHS Foundation Trust, Cambridge, UK
9 Medical Research Council Cognition and Brain Sciences Unit, Cambridge, United Kingdom
10 Clinical Neurochemistry Laboratory, Sahlgrenska University Hospital, Mölndal, S-431 80, Sweden
11 Department of Neurodegenerative Disease, UCL Institute of Neurology, London, WC1N 6BG, UK
12 UK Dementia Research Institute at UCL, London, WC1N 6BG, UK
13 Hong Kong Center for Neurodegenerative Diseases, Clear Water Bay, Hong Kong, China
14 Wisconsin Alzheimer's Disease Research Center, University of Wisconsin School of Medicine and Public Health, University of Wisconsin-Madison, Madison, WI, 53792, USA

*Joint Senior authors

Short running title: Blood inflammation markers in FTLD

Corresponding Author:

Dr. Maura Malpetti

Department of Clinical Neurosciences
University of Cambridge
Herchel Smith Building, Forvie Site
Robinson Way, Cambridge Biomedical Campus
Cambridge CB2 0SZ

Email: mm2243@medschl.cam.ac.uk

Supplementary material

Supplementary Table 1. Details of Cytokine Assays.

Cytokine	MSD Panel	Unit	LLOD	Source of LLOD	Included in analysis
IFN-γ	MSD V-plex Proinflammatory Panel 1	pg/ml	2.12	Calculated by MSD WorkBench software as part of the data interpretation from the analysis for this study	1
IL-10	MSD V-plex Proinflammatory Panel 1	pg/ml	0.24		0
IL-12p70	MSD V-plex Proinflammatory Panel 1	pg/ml	0.26		0
IL-13	MSD V-plex Proinflammatory Panel 1	pg/ml	1.96		0
IL-1β	MSD V-plex Proinflammatory Panel 1	pg/ml	1.4		0
IL-2	MSD V-plex Proinflammatory Panel 1	pg/ml	0.47		0
IL-4	MSD V-plex Proinflammatory Panel 1	pg/ml	0.07		0
IL-6	MSD V-plex Proinflammatory Panel 1	pg/ml	0.38		1
IL-8	MSD V-plex Proinflammatory Panel 1	pg/ml	0.26		1
TNF-α	MSD V-plex Proinflammatory Panel 1	pg/ml	0.58		1
Eotaxin	MSD V-plex Chemokine Panel 1	pg/ml	60		1
Eotaxin-3	MSD V-plex Chemokine Panel 1	pg/ml	9.87		1
IP-10	MSD V-plex Chemokine Panel 1	pg/ml	1.28		1
MCP-1	MSD V-plex Chemokine Panel 1	pg/ml	0.88		1
MCP-4	MSD V-plex Chemokine Panel 1	pg/ml	20.51		1
MDC	MSD V-plex Chemokine Panel 1	pg/ml	29.12		1
MIP-1α	MSD V-plex Chemokine Panel 1	pg/ml	25.01		0
MIP-1β	MSD V-plex Chemokine Panel 1	pg/ml	3.48		1
TARC	MSD V-plex Chemokine Panel 1	pg/ml	11.8		1
GM-CSF	MSD V-plex Cytokine Panel 1	pg/ml	0.67		0
IL-1α	MSD V-plex Cytokine Panel 1	pg/ml	0.82		0
IL-12	MSD V-plex Cytokine Panel 1	pg/ml	1.2		1
IL-15	MSD V-plex Cytokine Panel 1	pg/ml	0.42		1
IL-16	MSD V-plex Cytokine Panel 1	pg/ml	5.7		1
IL-17A	MSD V-plex Cytokine Panel 1	pg/ml	2.6		1
IL-5	MSD V-plex Cytokine Panel 1	pg/ml	0.5		0
IL-7	MSD V-plex Cytokine Panel 1	pg/ml	0.94		1
TNF-β	MSD V-plex Cytokine Panel 1	pg/ml	0.48		0
VEGF	MSD V-plex Cytokine Panel 1	pg/ml	0.78		1
IL- 17A	MSD V-plex TH17 Panel 1	pg/ml	14.62		0
IL-21	MSD V-plex TH17 Panel 1	pg/ml	19		0
IL-31	MSD V-plex TH17 Panel 1	pg/ml	0.8		0
IL-27	MSD V-plex TH17 Panel 1	pg/ml	66.96		1
IL-23	MSD V-plex TH17 Panel 1	pg/ml	13.04		0
IL-22	MSD V-plex TH17 Panel 1	pg/ml	1.35		0
MIP-3α	MSD V-plex TH17 Panel 1	pg/ml	4.58		1
IL-34	MSD R-plex	pg/ml	1.34		1
TNF-R1	MSD R-plex	pg/ml	3.8		1

M-CSF	MSD U-plex	pg/ml	0.76		1
YKL-40	MSD U-plex	pg/ml	2186		1
hsCRP	Dimension	mg/L	0.1	Manufacturer	1

Abbreviations: LLOD=Lower Limit of Detection; MSD= MesoScale Discovery

Supplementary Table 2. Dunn's Multiple Comparison post hoc test following the Kruskal-Wallis test on cytokine-derived Component 1.

Group 1	Group 2	n1	n2	statistic	p	p FDR
HC	bvFTD	29	52	3.119	0.002*	0.019*
HC	svPPA	29	20	1.715	0.086	0.259
HC	nfPPA	29	31	3.020	0.003*	0.019*
HC	PSP	29	58	2.316	0.021*	0.077
HC	CBS	29	53	2.784	0.005*	0.027*
bvFTD	svPPA	52	20	-0.852	0.394	0.657
bvFTD	nfPPA	52	31	0.252	0.801	0.858
bvFTD	PSP	52	58	-1.028	0.304	0.612
bvFTD	CBS	52	53	-0.409	0.683	0.788
svPPA	nfPPA	20	31	0.981	0.326	0.612
svPPA	PSP	20	58	0.108	0.914	0.914
svPPA	CBS	20	53	0.550	0.582	0.727
nfPPA	PSP	31	58	-1.139	0.255	0.612
nfPPA	CBS	31	53	-0.606	0.544	0.727
PSP	CBS	58	53	0.613	0.540	0.727

Abbreviations: bvFTD=behavioural variant frontotemporal dementia; nfPPA=non-fluent primary progressive aphasia; svPPA=semantic variant primary progressive aphasia; PSP=progressive supranuclear palsy; CBS= corticobasal syndrome; FDR=False Discovery Rate correction

Supplementary Table 3. Loadings from cytokine variables (red) and regional TSPO PET values (yellow) on the first CCA component.

Category	Variable	Loadings (contribution to CCA Component)
Cytokines	TNF-a	0.337
Cytokines	TNF-R1	0.325
Cytokines	M-CSF	0.305
Cytokines	IL-17A	0.284
Cytokines	IL-12	0.271
Cytokines	IP-10	0.257
Cytokines	IL-6	0.246
Cytokines	hsCRP	0.233
Cytokines	YKL-40	0.224
Cytokines	MDC	0.212
Cytokines	MIP-3a	0.204
Cytokines	IL-27	0.178
Cytokines	IFN-y	0.178
Cytokines	IL-16	0.178
Cytokines	Eotaxin	0.155
Cytokines	MIP-1b	0.155
Cytokines	MCP-4	0.129
Cytokines	IL-8	0.129
Cytokines	TARC	0.115
Cytokines	IL-34	0.114
Cytokines	VEGF	0.084
Cytokines	MCP-1	0.073
Cytokines	IL-15	0.041
Cytokines	Eotaxin-3	0.012
Cytokines	IL-7	-0.001
TSPO PET	Brainstem mid	0.387
TSPO PET	Brainstem pon	0.400
TSPO PET	Brainstem med	0.464
TSPO PET	FL mid fr G L	0.405
TSPO PET	FL precen G L	0.686
TSPO PET	FL strai G L	-0.030
TSPO PET	FL OFC AOG L	0.266
TSPO PET	FL inf fr G L	0.400
TSPO PET	FL sup fr G L	0.397
TSPO PET	FL OFC MOG L	0.275
TSPO PET	FL OFC LOG L	0.393
TSPO PET	FL OFC POG L	0.122
TSPO PET	Subgen antCing L	-0.244
TSPO PET	Subcall area L	-0.167

TSPO PET	Presubgen antCing L	-0.082
TSPO PET	FL mid fr G R	0.378
TSPO PET	FL precen G R	0.621
TSPO PET	FL strai G R	0.014
TSPO PET	FL OFC AOG R	0.225
TSPO PET	FL inf fr G R	0.458
TSPO PET	FL sup fr G R	0.432
TSPO PET	FL OFC MOG R	0.149
TSPO PET	FL OFC LOG R	0.388
TSPO PET	FL OFC POG R	0.277
TSPO PET	Subgen antCing R	-0.107
TSPO PET	Subcall area R	-0.296
TSPO PET	Presubgen antCing R	-0.043
TSPO PET	Hippocampus L	-0.094
TSPO PET	Amygdala L	0.004
TSPO PET	Ant TL med L	-0.109
TSPO PET	Ant TL inf Lat L	-0.104
TSPO PET	G paraH amb L	-0.123
TSPO PET	G sup temp cent L	0.314
TSPO PET	G tem midin L	-0.101
TSPO PET	G occtem La L	-0.154
TSPO PET	PosteriorTL L	0.216
TSPO PET	G sup temp ant L	-0.080
TSPO PET	Hippocampus R	0.081
TSPO PET	Amygdala R	-0.085
TSPO PET	Ant TL med R	-0.019
TSPO PET	Ant TL inf Lat R	-0.046
TSPO PET	G paraH amb R	0.057
TSPO PET	G sup temp cent R	0.355
TSPO PET	G tem midin R	0.076
TSPO PET	G occtem La R	0.232
TSPO PET	PosteriorTL R	0.428
TSPO PET	G sup temp ant R	-0.121
TSPO PET	PL Rest L	0.387
TSPO PET	PL postce G L	0.511
TSPO PET	PL sup pa G L	0.503
TSPO PET	PL Rest R	0.497
TSPO PET	PL postce G R	0.563
TSPO PET	PL sup pa G R	0.488
TSPO PET	Insula L	-0.026
TSPO PET	G cing ant sup L	0.186
TSPO PET	G cing post L	0.130
TSPO PET	Insula R	0.292

TSPO PET	G cing ant sup R	0.301
TSPO PET	G cing post R	0.370
TSPO PET	Corp Callosum	0.218
TSPO PET	CaudateNucl L	0.004
TSPO PET	NuclAccumb L	-0.180
TSPO PET	Putamen L	-0.045
TSPO PET	Thalamus L	0.059
TSPO PET	Pallidum L	0.132
TSPO PET	S nigra L	0.163
TSPO PET	CaudateNucl R	-0.113
TSPO PET	NuclAccumb R	-0.329
TSPO PET	Putamen R	0.014
TSPO PET	Thalamus R	0.064
TSPO PET	Pallidum R	0.196
TSPO PET	S nigra R	0.043
TSPO PET	OL Rest Lat L	0.343
TSPO PET	OL Ling G L	0.283
TSPO PET	OL cuneus L	0.211
TSPO PET	OL Rest Lat R	0.507
TSPO PET	OL Ling G R	0.329
TSPO PET	OL cuneus R	0.418
TSPO PET	Cerebellum gm L	0.225
TSPO PET	Cerebellum wm L	0.405
TSPO PET	Cerebellum dentate L	0.194
TSPO PET	Cerebellum gm R	0.199
TSPO PET	Cerebellum wm R	0.447
TSPO PET	Cerebellum dentate R	0.053
TSPO PET	Third Ventricl	-0.092
TSPO PET	Frontal Horn L	-0.057
TSPO PET	Tempora Horn L	-0.094
TSPO PET	Frontal Horn R	0.012
TSPO PET	Tempora Horn R	-0.063

Supplementary Figure 1. Pathways analysis of the leading cytokines in Component 1. Protein-protein interactions defined by the STRING software (https://string-db.org/), which systematically collects and integrates protein-protein physical interactions and functional associations. The analysis was run on lead cytokines from Component 1 (with contribution values > 0.4) and based on previous homo sapiens studies. The edges indicate both functional and physical protein associations, while the line thickness indicates the confidence and strength of data support.

Original Nomenclature	STRING Nomenclature
TNF-α	TNF
TNF-R1	TNFRSF1A
M-CSF	CSF1
IL-17A	IL17A
IL-12	IL12A
IP-10	CXCL10
IL-6	IL6
hsCRP	CRP
YKL-40	CHI3L1
MDC	CCL22
MIP-3α	CCL20
IFN-γ	IFNG
IL-27	IL27
IL-16	IL16

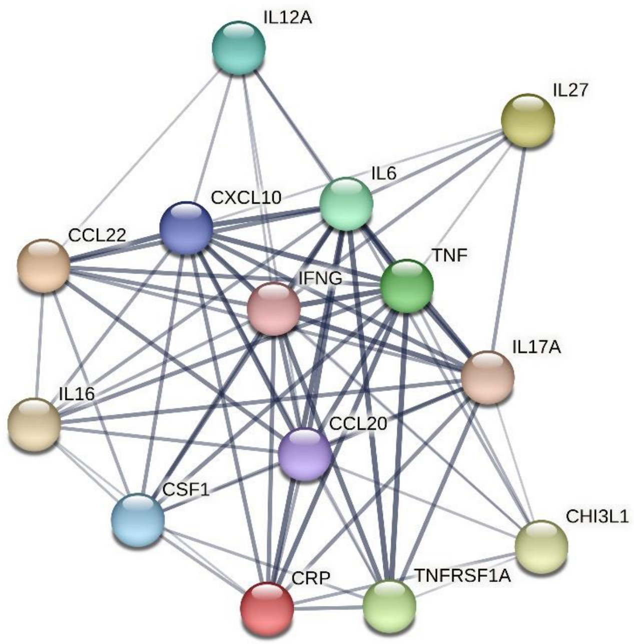

Supplementary Figure 2. Cytokine-derived principal components (PC) and correlation on individual scores obtained from the full cohort (n=243) and the reduced cohort (n=226) after excluding participants with hsCRP > 10 mg/L.

	PC 1 (n=243)
TNF-α	0.779
TNF-R1	0.743
M-CSF	0.702
IL-17A	0.661
IL-12	0.628
IP-10	0.601
IL-6	0.573
hsCRP	0.546
YKL-40	0.520
MDC	0.494
MIP-3α	0.479
IFN-γ	0.412
IL-27	0.411
IL-16	0.410
Eotaxin	0.360
MIP-1β	0.358
MCP-4	0.303
IL-8	0.298
TARC	0.271
IL-34	0.260
VEGF	0.199
MCP-1	0.173
IL-15	0.094
Eotaxin-3	0.030
IL-7	-0.001

	PC 1 (n=226, hsCRP < 10)
TNF-α	0.762
TNF-R1	0.727
M-CSF	0.663
IL-12	0.630
IL-17A	0.628
IP-10	0.622
MDC	0.492
IL-6	0.486
YKL-40	0.456
hsCRP	0.455
Eotaxin	0.424
IL-16	0.413
IFN-γ	0.400
MIP-3α	0.396
MIP-1β	0.353
IL-27	0.333
IL-34	0.311
MCP-4	0.310
TARC	0.306
IL-8	0.259
MCP-1	0.253
VEGF	0.143
IL-15	0.091
Eotaxin-3	0.023
IL-7	-0.087

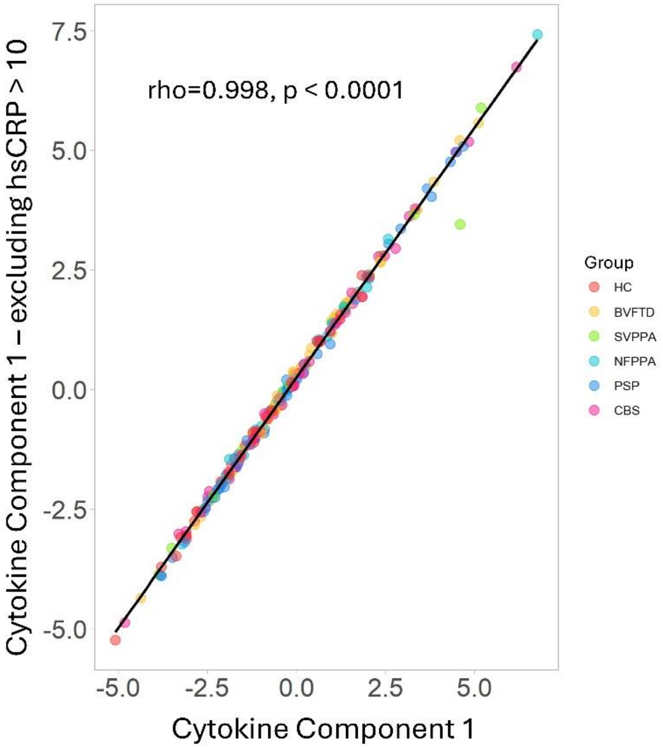

Supplementary Figure 3. Associations between individual loadings of cytokine-derived Component 1 and plasma marker levels.

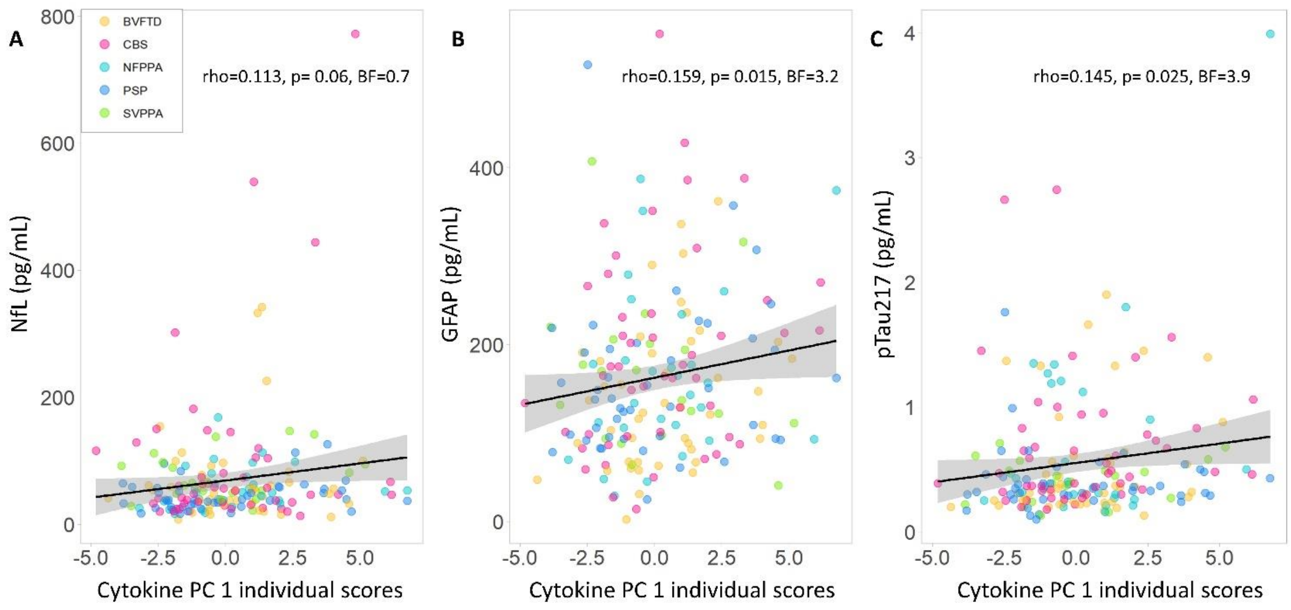

Supplementary Figure 4. Regions of interest (ROIs) for cytokine-PET correlations. ROI numbers and names refers to regional numbers and labels in the Hammersmith Atlas, while colours reflect Spearman correlation Rho as reported in Figure 4.

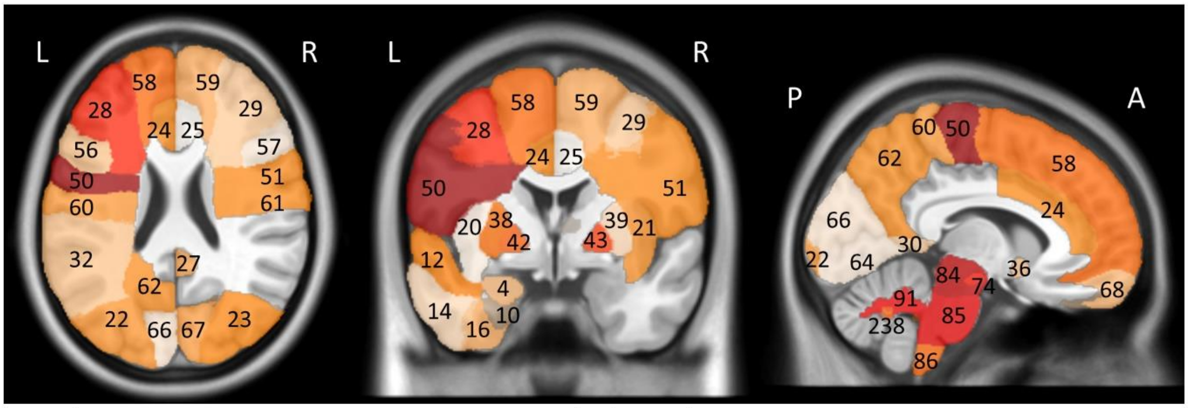

ROI #	ROI Name
4	Amygdala L
10	Parahippocampal and ambient gyri L
12	Superior temporal gyrus posterior part L
14	Middle and inferior temporal gyrus L
16	Fusiform gyrus L
20	Insula L
21	Insula R
22	Lateral remainder of occipital lobe L
23	Lateral remainder of occipital lobe R
24	Cingulate gyrus anterior part L
27	Gyrus cinguli posterior part R
28	Middle frontal gyrus L
29	Middle frontal gyrus R
32	Inferiolateral remainder of parietal lobe L
36	Nucleus accumbens L
38	Putamen L
39	Putamen R
42	Pallidum L
43	Pallidum R

ROI #	ROI Name
50	Precentral gyrus L
51	Precentral gyrus R
56	Inferior frontal gyrus L
57	Inferior frontal gyrus R
58	Superior frontal gyrus L
59	Superior frontal gyrus R
60	Postcentral gyrus L
61	Postcentral gyrus R
62	Superior parietal gyrus L
64	Lingual gyrus L
66	Cuneus L
67	Cuneus R
68	Medial orbital gyrus L
74	Substantia nigra L
84	Brainstem mid B
85	Brainstem pon B
86	Brainstem med B
91	Cerebellum wm L
238	Cerebellum dentate L
