# Supplementary material for: Blood inflammation relates to neuroinflammation and survival in frontotemporal lobar degeneration
Source: Brain. Author manuscript; Available in PMC 2024 Dec 27. (PMC7617268; doi:10.1093/brain/awae269)
Supplement: Supplementary file 1 [file EMS199602-supplement-Supplementary.docx]

**Blood inflammation relates to neuroinflammation and survival in frontotemporal lobar degeneration**

Maura Malpetti^1^, Peter Swann^2^, Kamen A Tsvetanov^1,2^, Leonidas Chouliaras^3^, Alexandra Strauss^1^, Tanatswa Chikaura^1^, Alexander G Murley^1^, Nicholas J. Ashton^4,5,6,7^, Peter Barker^8^, P Simon Jones^1^, Tim D. Fryer^1^, Young T. Hong^1^, Thomas E Cope^1,9^, George Savulich^2^, Duncan Street^1^, W Richard Bevan-Jones^2^, Timothy Rittman^1^, Kaj Blennow^4,10^, Henrik Zetterberg^4,10,11,12,13,14^, Franklin I. Aigbirhio^1^,  John T. O’Brien^2,*^ and James B. Rowe^1,9,*^

1 University of Cambridge Department of Clinical Neurosciences and Cambridge University Hospitals NHS Trust, Cambridge, United Kingdom  

2 Department of Psychology, University of Cambridge, Cambridge, United Kingdom 

3 Department of Psychiatry, University of Cambridge, Cambridge, United Kingdom 

4 Department of Psychiatry and Neurochemistry, Institute of Neuroscience and Physiology, The Sahlgrenska Academy, University of Gothenburg, Mölndal, S-431 80, Sweden

5 Wallenberg Centre for Molecular Medicine, University of Gothenburg, Gothenburg, S-413 45, Sweden

6 King’s College London, Institute of Psychiatry, Psychology and Neuroscience, Maurice Wohl Institute Clinical Neuroscience Institute, London, SE5 9RT, UK

7 NIHR Biomedical Research Centre for Mental Health and Biomedical Research Unit for Dementia at South London and Maudsley NHS Foundation, London, SE5 8AF, UK 

8 NIHR Cambridge Biomedical Research Centre, Core BiochemicalAssay Laboratory, Cambridge University Hospitals NHS Foundation Trust, Cambridge, UK 

9 Medical Research Council Cognition and Brain Sciences Unit, Cambridge, United Kingdom 

10 Clinical Neurochemistry Laboratory, Sahlgrenska University Hospital, Mölndal, S-431 80, Sweden

11 Department of Neurodegenerative Disease, UCL Institute of Neurology, London, WC1N 6BG, UK

12 UK Dementia Research Institute at UCL, London, WC1N 6BG, UK

13 Hong Kong Center for Neurodegenerative Diseases, Clear Water Bay, Hong Kong, China

14 Wisconsin Alzheimer’s Disease Research Center, University of Wisconsin School of Medicine and Public Health, University of Wisconsin-Madison, Madison, WI, 53792, USA

*Joint Senior authors

**Short running title:** Blood inflammation markers in FTLD

**Corresponding Author:**   

Dr. Maura Malpetti 

Department of Clinical Neurosciences 
University of Cambridge  
Herchel Smith Building, Forvie Site  
Robinson Way, Cambridge Biomedical Campus 
Cambridge CB2 0SZ 

Email: [mm2243@medschl.cam.ac.uk](mailto:mm2243@medschl.cam.ac.uk)  

**Supplementary material**

**Supplementary Table 1. Details of Cytokine Assays.**

| **Cytokine** | **MSD Panel** | **Unit** | **LLOD** | **Source of LLOD** | **Included in analysis** |
| --- | --- | --- | --- | --- | --- |
| IFN-γ | MSD V-plex Proinflammatory Panel 1 | pg/ml | 2.12 | Calculated by MSD WorkBench software as part of the data interpretation from the analysis for this study | 1 |
| IL-10 | MSD V-plex Proinflammatory Panel 1 | pg/ml | 0.24 |  | 0 |
| IL-12p70 | MSD V-plex Proinflammatory Panel 1 | pg/ml | 0.26 |  | 0 |
| IL-13 | MSD V-plex Proinflammatory Panel 1 | pg/ml | 1.96 |  | 0 |
| IL-1β | MSD V-plex Proinflammatory Panel 1 | pg/ml | 1.4 |  | 0 |
| IL-2 | MSD V-plex Proinflammatory Panel 1 | pg/ml | 0.47 |  | 0 |
| IL-4 | MSD V-plex Proinflammatory Panel 1 | pg/ml | 0.07 |  | 0 |
| IL-6 | MSD V-plex Proinflammatory Panel 1 | pg/ml | 0.38 |  | 1 |
| IL-8 | MSD V-plex Proinflammatory Panel 1 | pg/ml | 0.26 |  | 1 |
| TNF-α | MSD V-plex Proinflammatory Panel 1 | pg/ml | 0.58 |  | 1 |
| Eotaxin | MSD V-plex Chemokine Panel 1 | pg/ml | 60 |  | 1 |
| Eotaxin-3 | MSD V-plex Chemokine Panel 1 | pg/ml | 9.87 |  | 1 |
| IP-10 | MSD V-plex Chemokine Panel 1 | pg/ml | 1.28 |  | 1 |
| MCP-1 | MSD V-plex Chemokine Panel 1 | pg/ml | 0.88 |  | 1 |
| MCP-4 | MSD V-plex Chemokine Panel 1 | pg/ml | 20.51 |  | 1 |
| MDC | MSD V-plex Chemokine Panel 1 | pg/ml | 29.12 |  | 1 |
| MIP-1α | MSD V-plex Chemokine Panel 1 | pg/ml | 25.01 |  | 0 |
| MIP-1β | MSD V-plex Chemokine Panel 1 | pg/ml | 3.48 |  | 1 |
| TARC | MSD V-plex Chemokine Panel 1 | pg/ml | 11.8 |  | 1 |
| GM-CSF | MSD V-plex Cytokine Panel 1 | pg/ml | 0.67 |  | 0 |
| IL-1α | MSD V-plex Cytokine Panel 1 | pg/ml | 0.82 |  | 0 |
| IL-12 | MSD V-plex Cytokine Panel 1 | pg/ml | 1.2 |  | 1 |
| IL-15 | MSD V-plex Cytokine Panel 1 | pg/ml | 0.42 |  | 1 |
| IL-16 | MSD V-plex Cytokine Panel 1 | pg/ml | 5.7 |  | 1 |
| IL-17A | MSD V-plex Cytokine Panel 1 | pg/ml | 2.6 |  | 1 |
| IL-5 | MSD V-plex Cytokine Panel 1 | pg/ml | 0.5 |  | 0 |
| IL-7 | MSD V-plex Cytokine Panel 1 | pg/ml | 0.94 |  | 1 |
| TNF-β | MSD V-plex Cytokine Panel 1 | pg/ml | 0.48 |  | 0 |
| VEGF | MSD V-plex Cytokine Panel 1 | pg/ml | 0.78 |  | 1 |
| IL- 17A | MSD V-plex TH17 Panel 1 | pg/ml | 14.62 |  | 0 |
| IL-21 | MSD V-plex TH17 Panel 1 | pg/ml | 19 |  | 0 |
| IL-31 | MSD V-plex TH17 Panel 1 | pg/ml | 0.8 |  | 0 |
| IL-27 | MSD V-plex TH17 Panel 1 | pg/ml | 66.96 |  | 1 |
| IL-23 | MSD V-plex TH17 Panel 1 | pg/ml | 13.04 |  | 0 |
| IL-22 | MSD V-plex TH17 Panel 1 | pg/ml | 1.35 |  | 0 |
| MIP-3α | MSD V-plex TH17 Panel 1 | pg/ml | 4.58 |  | 1 |
| IL-34 | MSD R-plex | pg/ml | 1.34 |  | 1 |
| TNF-R1 | MSD R-plex | pg/ml | 3.8 |  | 1 |
| M-CSF | MSD U-plex | pg/ml | 0.76 |  | 1 |
| YKL-40 | MSD U-plex | pg/ml | 2186 |  | 1 |
| hsCRP | Dimension | mg/L | 0.1 | Manufacturer | 1 |

*Abbreviations: LLOD=Lower Limit of Detection; MSD= MesoScale Discovery*

**Supplementary Table 2. Dunn’s Multiple Comparison post hoc test following the Kruskal-Wallis test on cytokine-derived Component 1.**

| **Group 1** | **Group 2** | **n1** | **n2** | **statistic** | **p** | **p FDR** |
| --- | --- | --- | --- | --- | --- | --- |
| HC | bvFTD | 29 | 52 | 3.119 | 0.002* | 0.019* |
| HC | svPPA | 29 | 20 | 1.715 | 0.086 | 0.259 |
| HC | nfPPA | 29 | 31 | 3.020 | 0.003* | 0.019* |
| HC | PSP | 29 | 58 | 2.316 | 0.021* | 0.077 |
| HC | CBS | 29 | 53 | 2.784 | 0.005* | 0.027* |
| bvFTD | svPPA | 52 | 20 | -0.852 | 0.394 | 0.657 |
| bvFTD | nfPPA | 52 | 31 | 0.252 | 0.801 | 0.858 |
| bvFTD | PSP | 52 | 58 | -1.028 | 0.304 | 0.612 |
| bvFTD | CBS | 52 | 53 | -0.409 | 0.683 | 0.788 |
| svPPA | nfPPA | 20 | 31 | 0.981 | 0.326 | 0.612 |
| svPPA | PSP | 20 | 58 | 0.108 | 0.914 | 0.914 |
| svPPA | CBS | 20 | 53 | 0.550 | 0.582 | 0.727 |
| nfPPA | PSP | 31 | 58 | -1.139 | 0.255 | 0.612 |
| nfPPA | CBS | 31 | 53 | -0.606 | 0.544 | 0.727 |
| PSP | CBS | 58 | 53 | 0.613 | 0.540 | 0.727 |

*Abbreviations: bvFTD=behavioural variant frontotemporal dementia; nfPPA=non-fluent primary progressive aphasia; svPPA=semantic variant primary progressive aphasia; PSP=progressive supranuclear palsy; CBS= corticobasal syndrome; FDR=False Discovery Rate correction* 

**Supplementary Table 3. Loadings from cytokine variables (red) and regional TSPO PET values (yellow) on the first CCA component.**

| **Category** | **Variable** | **Loadings (contribution to CCA Component)** |
| --- | --- | --- |
| Cytokines | TNF-a | 0.337 |
| Cytokines | TNF-R1 | 0.325 |
| Cytokines | M-CSF | 0.305 |
| Cytokines | IL-17A | 0.284 |
| Cytokines | IL-12 | 0.271 |
| Cytokines | IP-10 | 0.257 |
| Cytokines | IL-6 | 0.246 |
| Cytokines | hsCRP | 0.233 |
| Cytokines | YKL-40 | 0.224 |
| Cytokines | MDC | 0.212 |
| Cytokines | MIP-3a | 0.204 |
| Cytokines | IL-27 | 0.178 |
| Cytokines | IFN-y | 0.178 |
| Cytokines | IL-16 | 0.178 |
| Cytokines | Eotaxin | 0.155 |
| Cytokines | MIP-1b | 0.155 |
| Cytokines | MCP-4 | 0.129 |
| Cytokines | IL-8 | 0.129 |
| Cytokines | TARC | 0.115 |
| Cytokines | IL-34 | 0.114 |
| Cytokines | VEGF | 0.084 |
| Cytokines | MCP-1 | 0.073 |
| Cytokines | IL-15 | 0.041 |
| Cytokines | Eotaxin-3 | 0.012 |
| Cytokines | IL-7 | -0.001 |
| TSPO PET | Brainstem mid | 0.387 |
| TSPO PET | Brainstem pon | 0.400 |
| TSPO PET | Brainstem med | 0.464 |
| TSPO PET | FL mid fr G L | 0.405 |
| TSPO PET | FL precen G L | 0.686 |
| TSPO PET | FL strai G L | -0.030 |
| TSPO PET | FL OFC AOG L | 0.266 |
| TSPO PET | FL inf fr G L | 0.400 |
| TSPO PET | FL sup fr G L | 0.397 |
| TSPO PET | FL OFC MOG L | 0.275 |
| TSPO PET | FL OFC LOG L | 0.393 |
| TSPO PET | FL OFC POG L | 0.122 |
| TSPO PET | Subgen antCing L | -0.244 |
| TSPO PET | Subcall area L | -0.167 |
| TSPO PET | Presubgen antCing L | -0.082 |
| TSPO PET | FL mid fr G R | 0.378 |
| TSPO PET | FL precen G R | 0.621 |
| TSPO PET | FL strai G R | 0.014 |
| TSPO PET | FL OFC AOG R | 0.225 |
| TSPO PET | FL inf fr G R | 0.458 |
| TSPO PET | FL sup fr G R | 0.432 |
| TSPO PET | FL OFC MOG R | 0.149 |
| TSPO PET | FL OFC LOG R | 0.388 |
| TSPO PET | FL OFC POG R | 0.277 |
| TSPO PET | Subgen antCing R | -0.107 |
| TSPO PET | Subcall area R | -0.296 |
| TSPO PET | Presubgen antCing R | -0.043 |
| TSPO PET | Hippocampus L | -0.094 |
| TSPO PET | Amygdala L | 0.004 |
| TSPO PET | Ant TL med L | -0.109 |
| TSPO PET | Ant TL inf Lat L | -0.104 |
| TSPO PET | G paraH amb L | -0.123 |
| TSPO PET | G sup temp cent L | 0.314 |
| TSPO PET | G tem midin L | -0.101 |
| TSPO PET | G occtem La L | -0.154 |
| TSPO PET | PosteriorTL L | 0.216 |
| TSPO PET | G sup temp ant L | -0.080 |
| TSPO PET | Hippocampus R | 0.081 |
| TSPO PET | Amygdala R | -0.085 |
| TSPO PET | Ant TL med R | -0.019 |
| TSPO PET | Ant TL inf Lat R | -0.046 |
| TSPO PET | G paraH amb R | 0.057 |
| TSPO PET | G sup temp cent R | 0.355 |
| TSPO PET | G tem midin R | 0.076 |
| TSPO PET | G occtem La R | 0.232 |
| TSPO PET | PosteriorTL R | 0.428 |
| TSPO PET | G sup temp ant R | -0.121 |
| TSPO PET | PL Rest L | 0.387 |
| TSPO PET | PL postce G L | 0.511 |
| TSPO PET | PL sup pa G L | 0.503 |
| TSPO PET | PL Rest R | 0.497 |
| TSPO PET | PL postce G R | 0.563 |
| TSPO PET | PL sup pa G R | 0.488 |
| TSPO PET | Insula L | -0.026 |
| TSPO PET | G cing ant sup L | 0.186 |
| TSPO PET | G cing post L | 0.130 |
| TSPO PET | Insula R | 0.292 |
| TSPO PET | G cing ant sup R | 0.301 |
| TSPO PET | G cing post R | 0.370 |
| TSPO PET | Corp Callosum | 0.218 |
| TSPO PET | CaudateNucl L | 0.004 |
| TSPO PET | NuclAccumb L | -0.180 |
| TSPO PET | Putamen L | -0.045 |
| TSPO PET | Thalamus L | 0.059 |
| TSPO PET | Pallidum L | 0.132 |
| TSPO PET | S nigra L | 0.163 |
| TSPO PET | CaudateNucl R | -0.113 |
| TSPO PET | NuclAccumb R | -0.329 |
| TSPO PET | Putamen R | 0.014 |
| TSPO PET | Thalamus R | 0.064 |
| TSPO PET | Pallidum R | 0.196 |
| TSPO PET | S nigra R | 0.043 |
| TSPO PET | OL Rest Lat L | 0.343 |
| TSPO PET | OL Ling G L | 0.283 |
| TSPO PET | OL cuneus L | 0.211 |
| TSPO PET | OL Rest Lat R | 0.507 |
| TSPO PET | OL Ling G R | 0.329 |
| TSPO PET | OL cuneus R | 0.418 |
| TSPO PET | Cerebellum gm L | 0.225 |
| TSPO PET | Cerebellum wm L | 0.405 |
| TSPO PET | Cerebellum dentate L | 0.194 |
| TSPO PET | Cerebellum gm R | 0.199 |
| TSPO PET | Cerebellum wm R | 0.447 |
| TSPO PET | Cerebellum dentate R | 0.053 |
| TSPO PET | Third Ventricl | -0.092 |
| TSPO PET | Frontal Horn L | -0.057 |
| TSPO PET | Tempora Horn L | -0.094 |
| TSPO PET | Frontal Horn R | 0.012 |
| TSPO PET | Tempora Horn R | -0.063 |

**Supplementary Figure 1. Pathways analysis of the leading cytokines in Component 1.** Protein-protein interactions defined by the STRING software ([https://string-db.org/](https://string-db.org/)), which systematically collects and integrates protein-protein physical interactions and functional associations. The analysis was run on lead cytokines from Component 1 (with contribution values > 0.4) and based on previous homo sapiens studies. The edges indicate both functional and physical protein associations, while the line thickness indicates the confidence and strength of data support.

**Supplementary Figure 2. Cytokine-derived principal components (PC) and correlation on individual scores obtained from the full cohort (n=243) and the reduced cohort (n=226) after excluding participants with hsCRP > 10 mg/L.**

**Supplementary Figure 3. Associations between individual loadings of cytokine-derived Component 1 and plasma marker levels.**

**Supplementary Figure 4. Regions of interest (ROIs) for cytokine-PET correlations.** ROI numbers and names refers to regional numbers and labels in the Hammersmith Atlas, while colours reflect Spearman correlation Rho as reported in Figure 4.
